# Higher levels of D2R and D3R in the frontal–striatal regions are associated with reduced perseverative reward seeking after opioid abstinence

**DOI:** 10.3389/fnbeh.2025.1552055

**Published:** 2025-06-02

**Authors:** Yingying Li, Xigeng Zheng, Cong Gao, Shao Li, Zhengkui Liu, Meixuan Lv, Fei Xiao, Yunjing Bai

**Affiliations:** ^1^State Key Laboratory of Cognitive Science and Mental Health, Institute of Psychology, Chinese Academy of Sciences, Beijing, China; ^2^Department of Psychology, University of Chinese Academy of Sciences, Beijing, China; ^3^Department of Physiology, College of Basic Medical Sciences, Liaoning Provincial Key Laboratory of Cerebral Diseases, National-Local Joint Engineering Research Center for Drug-Research and Development (R&D) of Neurodegenerative Diseases, Dalian Medical University, Dalian, China

**Keywords:** morphine, reward seeking, perseveration, D2/D3, striatum, prefrontal cortex

## Abstract

**Introduction:**

The lower levels of dopamine D2 receptor (D2R) in the striatum and the heightened levels of dopamine D2 receptor (D3R) in the midbrain have been linked to impulsive behavior and risky decision-making associated with drug dependence. While D3R has been considered a potential target for treating drug dependence, the connection between D3R in the prefrontal-striatal regions and maladaptive drug-related behaviors remains poorly understood.

**Methods:**

This study utilized two high-cost tasks to investigate perseverative reward seeking, specifically conflict-based approaching behavior and persistent responding behavior under a progesterone receptor (PR) procedure. Additionally, D2R and D3R levels in the medial prefrontal cortex (mPFC) and striatum were examined through Western blotting.

**Results:**

After each task, male rats were divided into two subpopulations: high-approaching vs. low-approaching and high-responding vs. low-responding. Rats treated with morphine (MOR) exhibited a 3 fold increase in the likelihood of developing high-approaching or high-responding behaviors compared to drug-naïve rats. D2R expression was higher in the ventral striatum of morphine-treated, low-approaching rats than high-approaching rats, negatively correlating with approaching behaviors within the morphine-exposed group. After six consecutive PR sessions, D3R levels in the dorsal striatum differed significantly between morphine-treated, low-responding rats and morphine-treated, high-responding rats, negatively correlating with responding behaviors within the morphine-exposed group. An intriguing finding was the non-linear relationships, resembling an inverted U shape, observed between the level of D3R in the mPFC and reward-seeking behaviors, as revealed by both tasks.

**Discussion:**

The elevated or relatively higher levels of D2R and D3R in the frontal-striatal regions may serve as protective factors for individuals abstaining from opioids, enabling them to control their reward-seeking behavior better.

## 1 Introduction

Individuals dependent on substances often exhibit deficits in inhibitory control, leading to inflexible, impulsive, or perseverative behaviors that are closely associated with compulsivity and relapse risk (Ersche et al., 2008; Noël et al., 2013; Jentsch and Pennington, 2014; Smith et al., 2014; Loree et al., 2014). Preclinical studies have also shown deficits in behavioral inhibition and flexibility in animals exposed to dependence-producing drugs (Jentsch et al., 2002; Bai et al., 2014; Gass et al., 2014; Li et al., 2017; Groman et al., 2017, 2020a), suggesting that the dysfunction of inhibitory control in dependent individuals is, in part, a consequence of drug exposure.

The drug-induced dysfunction of frontal–striatal circuits is critically involved in behavioral disinhibition (Moorman and Aston-Jones, 2015; Morein-Zamir and Robbins, 2015; Meyer and Bucci, 2016). Impaired dopamine signaling in these circuits, partly due to alterations in dopamine D2 receptors (D2Rs or D2/3Rs), plays an important role, since low levels of D2Rs have consistently been observed in the striatum of drug-dependent humans (Volkow et al., 2004; Briand et al., 2008; Fehr et al., 2008; Lee et al., 2009) and animals exposed to dependence-producing drugs (Spangler et al., 2003; Nader et al., 2008; Conrad et al., 2010; Tacelosky et al., 2015). Previous studies have demonstrated a link between low striatal D2R availability or expression and different facets of impulsivity in both drug-naïve animals and amphetamine-dependent humans (Dalley et al., 2007; Simon et al., 2013; Barlow et al., 2018; Lee et al., 2009; Ballard et al., 2015). Low striatal D2R binding predicts increased alcohol craving, which correlates with the high relapse risk in alcoholics (Heinz et al., 2004, 2005). Reduced expression of D2R in the ventral striatum has been correlated with greater heroin seeking in rats (Tacelosky et al., 2015), highlighting the importance of D2Rs in drug-related behavioral processes.

In contrast to D2R, recent research has reported heightened levels of the D3 receptor, a member of the D2-like receptor family (Sokoloff et al., 1990), in the brains of stimulant-dependent humans and drug-exposed rodents. Although there is some controversy (Chukwueke et al., 2021), several PET studies using the D3R-preferring ligand ([11C]-(+)-PHNO) have found increased D3R availability in the midbrain of methamphetamine and cocaine users, particularly in the substantia nigra (Boileau et al., 2012; Payer et al., 2013; Boileau et al., 2015). Heightened D3R levels in the midbrain have been associated with impulsive/risky decision-making in cocaine-dependent individuals and inflexible decision-making and susceptibility to cocaine use in rats (Payer et al., 2013; Groman et al., 2016, 2020b). However, the drug-induced alterations of D3R in the limbic forebrain, including the striatum, where D2R expression is highly abundant, remain unclear (Boileau et al., 2012; Payer et al., 2013; Worhunsky et al., 2017; Chukwueke et al., 2021). Additionally, while quite many preclinical studies have reported increased D3R binding, mRNA, and protein expression in the striatum following exposure to drugs such as cocaine, morphine (MOR), alcohol, or nicotine (Le Foll et al., 2002, 2003; Spangler et al., 2003; Neisewander et al., 2004; Vengeliene et al., 2006; Conrad et al., 2010; Collins et al., 2011), not all studies have yielded consistent results (Wallace et al., 1996; Chiang et al., 2003). Human postmortem studies have also observed higher levels of D3R binding or mRNA in the striatum of cocaine overdose fatalities (Staley and Mash, 1996; Segal et al., 1997; Mash and Staley, 1999), further suggesting the potential role of heightened D3R in drug-related processes. Several promising preclinical and clinical results support the utility of D3R antagonism as pharmacotherapy in drug dependence (Sokoloff and Le Foll, 2016; Galaj et al., 2018, 2020). However, the relationship between D3R alterations in the prefrontal cortex and striatum, pivotal regions for drug dependence, and dependence-related behaviors remains unknown.

Our previous work has shown that rats withdrawn from a binge-like morphine exposure exhibit two types of maladaptive behavior: persistent approaching behavior toward a sexual partner despite a continuously heightened obstacle and persistent operant responding for sucrose reward on a progressive ratio schedule (PR) of reinforcement (Bai et al., 2014, 2017; Li et al., 2017). These persistent reward-seeking behaviors, regardless of cost, suggest opioid-induced perseveration, inflexibility, or disinhibition of behavior in rats, potentially involving alterations in D2R and D3R levels within the frontal–striatal regions. Brain-derived neurotrophic factor (BDNF), required for the expression of D3R in some brain regions, may also participate in behavioral regulation by influencing D3R expression and dopamine responsiveness (Guillin et al., 2001). Therefore, the present study aimed to investigate concurrent alterations in D2R, D3R, and BDNF in the medial prefrontal cortex (mPFC) and striatum following opioid abstinence, and their relationships with maladaptive reward-seeking behaviors.

## 2 Materials and methods

**Figure 1:**
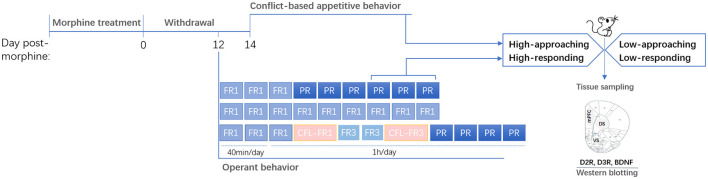
Experimental design. Rats were treated with and then withdrawn from morphine. Behavioral phenotypes (high- or low-approaching/responding) were characterized based on the appetitive behaviors or operant behaviors. Brain tissues were sampled after behavioral tests. mPFC, medial prefrontal cortex; DS, dorsal striatum; VS, ventral striatum.

The experimental design is shown in Figure 1.

### 2.1 Animals

Sprague–Dawley rats (Vital River Animal Center, Beijing, China; 155 male rats and 28 female rats in total) were housed in colony rooms with a controlled temperature (22–24°C) and humidity (40–60%) on a 12 h light/dark cycle. All procedures used in our experiment followed those described previously by Bai et al. (2017) and Li et al. (2017). They were conducted in accordance with the National Institutes of Health Guide for the Care and Use of Laboratory Animals (NIH Publications No. 8023, revised 1978).

### 2.2 Drugs

Morphine hydrochloride (Qinghai Pharmaceutical Co. Ltd, Qinghai, China) was dissolved in sterile physiological saline (SAL) at a final concentration of 20 mg/ml.

### 2.3 Binge-like morphine treatment

Male rats were treated twice daily for 5 days with intraperitoneal injections of either saline or morphine delivered in a binge-like regimen (Bai et al., 2014): 10, 20, 20, 40, 40, 40, 40, 40, 40, 40 mg/kg. The two doses of morphine administered on each day were at a time gap of approximately 6 h. Rats were returned to their home cage immediately after each injection. All rats underwent a withdrawal period of at least 14 days after the last saline or morphine administration (Bai et al., 2017, 2019).

### 2.4 Characterization of high- and low-approaching rats based on the conflict-based appetitive behaviors

#### 2.4.1 Animals

Twenty-eight male and 28 female rats (males weighing 250–300 g and females weighing 200–220 g on arrival) were housed four per cage (50 cm × 22.5 cm × 30 cm) with a reversed 12 h light/dark cycle (lights on at 21:00). Males weighed 330–400 g at the beginning of the experiments and females weighed 230–250 g upon ovariectomy. Females were bilaterally ovariectomized under 1% pentobarbital sodium (55 mg/kg, i.p.) anesthesia at least 2 weeks before use. Artificial estrus was induced by subcutaneous treatment with estradiol benzoate (25 μg/rat) and progesterone (1 mg/rat) about 48–52 and 4–6 h before tests, respectively, so that the female rats used for the conflict-based test were at the same stage of the cycle and highly receptive (Bai et al., 2014; Li et al., 2017; Bai et al., 2017). All tests were performed between 10:00 and 20:00 h during the dark phase of the cycle.

#### 2.4.2 Apparatus

**Figure 2:**
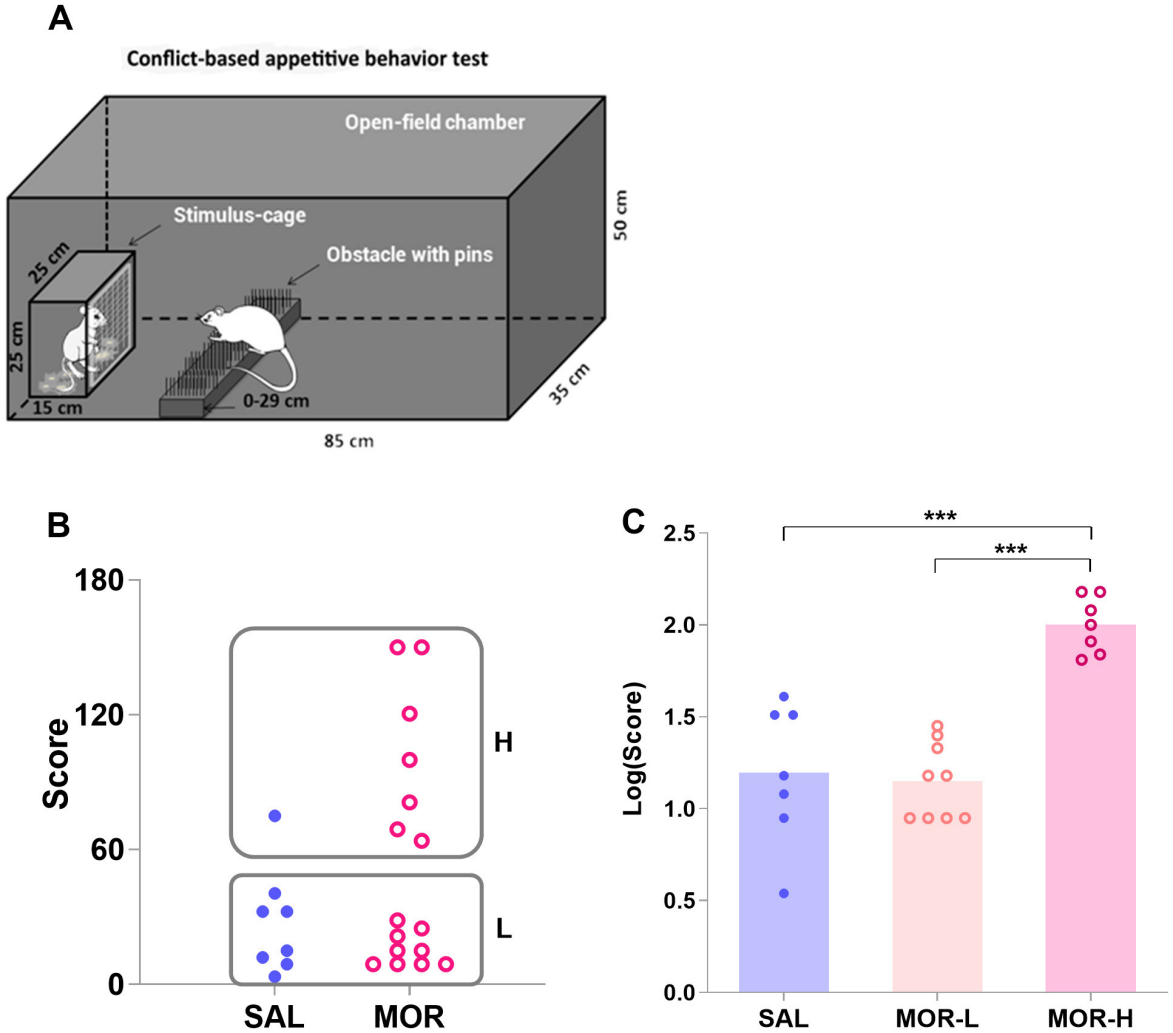
Characterization of the high- and low-approaching rats in the conflict-based test of appetitive behavior. The subjects in the open-field chamber had to surmount a dangerous obstacle, that is, climb over a continuously heightened board thick with pins, to approach the stimulus cage holding an estrous female rat **(A)**. **(B)** Shows the scores for approaching behavior in saline (SAL)- and morphine (MOR)-treated rats. Two subpopulations of rats were identified by a K-means cluster analysis of the scores: H, high-approaching; L, low-approaching. The logarithmic scores [Log(Score)] for approaching behavior in the subpopulations were compared (SAL-L, *n* = 7; SAL-H, *n* = 1; MOR-L, *n* = 9; MOR-H, *n* = 7) **(C)**. Data are expressed as data points of each rat **(B)** or mean ± SEM **(C)**. ***p < 0.001.

An open-field reward-proximity chamber made of black Plexiglas was used to assess the conflict-based appetitive behaviors for sexual reward (Figure 2A). A wire-screen stimulus cage (15 cm × 25 cm × 25 cm high) was mounted at one end of the open-field arena (85 cm × 35 cm × 50 cm high). The front of the cage was made of wire mesh (1-mm wire, mesh size: 10 mm × 10 mm), which allowed the male subjects to approach and investigate (i.e., to sniff) the estrous female rat in the stimulus cage but prevented physical contact with the female rat.

#### 2.4.3 Behavioral screening of experimental male rats

Male rats were screened for copulation under dim light during the dark phase of the cycle (13:00~19:00 h). Individual male rats were placed for a 5-min acclimation period in a box (60 cm × 50 cm × 40 cm height) with pine wood shaving bedding. Then, a receptive female rat was introduced, and male copulatory behaviors were monitored by experienced observers. The copulation on each day ended after the rat completed its first ejaculation within 30 min. Only those that performed successful ejaculation within 30 min for 3 consecutive days were assigned to the saline or morphine treatment group (24 male rats passed the screening).

#### 2.4.4 Conflict task procedure

Male rats pretreated with SAL (*n* = 8) or MOR (*n* = 16) underwent the conflict-based test for sexual reward (Bai et al., 2014; Li et al., 2017) on day 14 post-morphine treatment. We deliberately included a high number of rats in the morphine group because we anticipated that the morphine-treated rats would be further divided into high and low subgroups based on our previous studies. This experiment was performed during the dark phase (13:00~20:00). On the day before the test, all rats were habituated for 15 min to the open-field arena (without any obstacles). On the testing day, male rats were exposed for 10 min to the open-field arena prior to the introduction in the stimulus cage of a sexually receptive female and some female-soiled bedding (about 30 g) previously collected from one cage that had contained three sexually receptive females for 5 days (females replaced every day) and stored in the freezer until the day of the experiment. The male rats were then given 5 min to freely approach and investigate the sexual reinforcer, after which they were moved away from it, and the first trial of the test began with the insertion of an obstacle on the floor of the open field, 20 cm away from the wire screen of the stimulus cage. For the first trial, the obstacle consisted of a 14-cm wide, 3-mm thick board filled with pins (0.5 mm in diameter). With the test continuing, the obstacle became more and more difficult to surmount by replacing the board with pins of different features and repeatedly heightening the board. According to the length of, and the average distance between pins, three types of board were used: (a) length: 0.5 cm; average distance: 1 cm; (b) length: 0.8 cm; average distance: 0.5 cm; (c) Length: 2 cm; average distance: 1 cm. The board was repeatedly heightened as follows: 0, 2, 4, 7, 10, 13, 17, 21, 25, 29 cm. Thus, the 12-level difficulties of surmounting the obstacle, that is, 12 trials during the test were as follows: a + 0 cm, a + 2 cm, a + 4 cm, b + 4 cm, b + 7 cm, b + 10 cm, b + 13 cm, b + 17 cm, c + 17 cm, c + 21 cm, c + 25 cm, c + 29 cm. One trial was completed when the subject climbed or jumped over the obstacle 3 times within 4 min. Then the subsequent trial started. After surmounting the obstacle, the subject was moved away from the stimulus cage about 15–20 s. The test ended if the subject surmounted the obstacle < 3 times within 4 min. The amount of difficulty the subject overcame every time to approach the stimulus cage was graded and summed up to the total individual score as the measurement of approaching behavior (Table 1).

**Table 1:** Conflict-based appetitive behavior test.

**Trial**	**Amount of difficulty**	**Graded per approach**
1	a + 0 cm	0.5
2	a + 2 cm	1
3	a + 4 cm	1.5
4	b + 4 cm	3
5	b + 7 cm	3.5
6	b + 10 cm	4
7	b + 13 cm	4.5
8	b + 17 cm	5
9	c + 17 cm	6
10	c + 21 cm	6.5
11	c + 25 cm	7
12	c + 29 cm	7.5

“a”, “b,” and “c” represented three types of boards with different lengths and average distances between pins. (a) Length: 0.5 cm; average distance: 1 cm; (b) Length: 0.8 cm; average distance: 0.5 cm; (c) Length: 2 cm; average distance: 1 cm. “0, 2, … 25, 29 cm” indicated different heights of boards.

### 2.5 Characterization of high- and low-responding rats based on the PR performance

#### 2.5.1 Animals

One hundred and twenty-seven male rats weighing 300–340 g on arrival were individually housed in the home cages (25 cm × 22.5 cm × 30 cm) under a 12 h light-dark cycle (lights on at 07:00 h). All rats were familiarized with sucrose to avoid neophobia by giving them 48-h access to a bottle of 2.5% sucrose solution (w/v) in their home cages. Food was given *ad libitum* during this period. Bottle weights were recorded prior to and immediately after this familiarization period. The consumption of sucrose solutions by each rat was calculated as a function of body weight [the amount (g) of solution consumed per weight (100 g)]. Rats were randomly assigned to either the saline-treated or the morphine-treated group while maintaining equal amounts of sucrose consumption across the two groups.

#### 2.5.2 Apparatus

Instrumental responding for sucrose was measured in eight operant chambers (33 cm × 27 cm × 33 cm; AniLab Software and Instruments Co., Ltd., Ningbo, China). Each chamber was enclosed in a sound-attenuating box with a 50-dB background noise generated by the self-administration hardware. Each chamber was fitted with two nose-poke operandi (2.5 cm in diameter)—each of them located on the left or the right side of a central liquid receptacle. Two yellow LED cue lights (20 mW) were separately inside each nose-poke hole. A white cage light was fixed 20 cm above the right nose-poke. Sucrose solution was delivered through a metal spout attached to a 60-ml syringe pump with tubing that delivered fluid at 34.50-ml/min speed. The pumps were calibrated to dispense 0.08 ml in 0.139 s of the solution into a reinforced liquid receptacle in 0.139 s. A 10-s time-out period was initiated following each reinforcement so that subsequent responding produced no effect in this period.

#### 2.5.3 Operant behavior procedure

All the operant behavior experiments were performed during the light cycle (09:00~17:00). Rats were trained to respond for a 15% sucrose solution under continuous reinforcement (on fixed ratio 1 schedule, FR1) or respond for the same sucrose solution on a progressive ratio schedule (PR) of reinforcement as previously described (Bai et al., 2014). Briefly, on day 11 post-morphine treatment, all rats were allowed to habituate to the operant chambers for 15 min (without any light on), following which they were trained to learn the contingency between house light (as the stimulus) and the delivery of 0.08 ml water dispensed in the central liquid receptacle for 60 min. The chamber was continuously illuminated by two nose-poke lights, and the liquid was delivered into the central receptacle on a variable interval (40 s on average, ranging from 10 to 70 s) schedule independent of nose-poke behaviors (Chudasama and Robbins, 2003). Notably, 1 s before a liquid dropped, the house light was switched on for 4 s. Water and food were removed from home cages 12 and 18 h, respectively, before the habituation session.

On the following day, rats were trained to nose poke to obtain 0.08 ml of 15% sucrose under an FR1 schedule (Rossetti et al., 2013). Rats were considered to have acquired the task when they successfully obtained 60 reinforcers within one session over two successive days (40 min/session; one session a day). If they failed to achieve this performance within the allocated time, they were re-trained in an additional session after a 3- interval. Rats were excluded from the experiment if they failed to meet the criterion after this re-training session. Rats could be trained only for three sessions at most.

No rats dropped out of the training. Then, 25 saline-treated and 29 morphine-treated rats were subjected to daily sessions (1 h/session) under an FR1 schedule from day 14–20 post-morphine treatment, and 24 saline-treated and 32 morphine-treated rats were subjected to a FR1 session (1 h) on day 14 and to daily sessions (1 h/session) from day 15–20 post-morphine treatment under a PR schedule. Reinforcers were earned according to the following number of nose pokes: 1, 2, 4, 6, 9, 12, 15, 20, 25, 32, 40, 50, 62, 77, 95, 118, 145, … This number series was derived from the following equation (Richardson and Roberts, 1996):


$$\begin{array}{l} \text{Response ratio }(\text{rounded to}\;\\ \;\text{nearest integer})\text{= }\left[{\text{5e}}^{\left(reinforcer\;number\;\times \text{ 0}.\text{2}\right)}\right]\;-\text{5} \end{array}$$

The final response ratio achieved represented the “breaking point” value, which was adjusted as “reinforcers obtained” in the figures. The session ended either when rats failed to reach the following nose-poke criterion within 30 min, or when the session duration reached 1 h. The number of reinforcements obtained by each individual under FR1 and PR was recorded.

Throughout the operant training and testing phases, water was removed for 2 h before the daily session, and food was supplied for 1 h after each session ended to maintain a body weight above 85% of their baseline weight (Dias-Ferreira et al., 2009).

#### 2.5.4 Contrafreeloading (CFL) behavior

The same operant chambers and experimental procedures (as described above) were also used to investigate CFL behaviors of saline- and morphine-treated animals (SAL, *n* = 8, MOR, *n* = 9) and their relations to PR performance.

Rats were given free access to food for 1 h after each session and were water-restricted for 2 h before each session over the training and testing period. From day 13 post-morphine treatment, after a 15-min habituation period, all rats were trained to respond for 15% sucrose solution on an FR1 schedule for two 40-min daily sessions (as described above). On day 16 post-morphine treatment, CFL testing started with a bottle (identical to a water bottle in a home cage) filled with 15% sucrose solution mounted on the chamber's front wall. Hence, animals had access to two sources of sucrose reward simultaneously during 1-h CFL testing, that is, freely available in the bottle and upon responding under FR1. On the next 2 days, all rats were trained for 1 h to respond to a 15% sucrose solution on an FR3 schedule and then tested for CFL behavior for 1 h under FR3. The amount of sucrose solution ingested by animals was measured by weighing the bottle before and after each session and calculating the total volume of delivered sucrose solution in the magazine (number of reinforcements × 0.08 ml). The CFL level, a measure of perseveration/compulsivity, was calculated as the percentage of the fraction of total fluid intake gained instrumentally (delivered sucrose solution/total sucrose solution intake × 100; Milella et al., 2008; Frederick and Cocuzzo, 2017). From the day next to the last CFL testing, rats were subjected to four PR sessions (1 h/session/day) under food and water deprivation. The number of reinforcers obtained by each rat was recorded.

### 2.6 Western blotting

Rats were decapitated 30 min after the conflict task or the last PR session under anesthesia. Brains were harvested and instantly frozen in dry ice (−60°C) for 45 s and stored at −80°C. Punches were obtained from brain slices (coronal sections) no thicker than 300 μm with a puncher in a 3-mm outer diameter in a cryostat microtome. The ventral striatum (VS) and dorsal striatum (DS) were each collected bilaterally from Bregma +2.76 to +0.96 mm, ML ± 1.2 mm, DV 7 to 7.2 mm, and from Bregma +1.92 to + 0.00 mm, ML ±2.4 to ±2.8 mm, DV 5.2 to 5.6 mm. The mPFC, including the prelimbic and anterior cingulate cortices, was taken along the midline from Bregma +3.72 to +2.52 mm, ML ±0.6 mm, DV 2.8 to 3.2 mm (see Supplementary Figure). Samples from the mPFC, VS, and DS were then stored at −80°C until Western blotting assays.

An aliquot of brain sample from each rat was homogenized (15,000 rpm, 30 s) in a lysis buffer (50-mM Tris–HCl, pH 8.0; 150-mM NaCl; 1% NP-40 [Sigma-Aldrich (Shanghai) Trading Co. Ltd]; 1% sodium deoxycholate sulfate (SDS), 0.1%). Notably, 1-mM phenylmethanesulfonyl fluoride (PMSF) was added to the homogenate, which was placed on ice for 30 min, and then centrifuged (13,000 rpm) for 10 min. Quantification of total protein in the supernatant was performed with a bicinchoninic acid (BCA) kit (Pierce, Rockford, IL, USA). Samples (60-μg total protein) were diluted in electrophoresis sample buffer and loaded onto 10% sodium dodecyl sulfate-polyacrylamide gel (SDS-PAGE). After separation by SDS-PAGE, proteins were electroblotted onto polyvinylidene fluoride (PVDF) membranes and blocked with 5% non-fat dried milk in Tris-buffered saline with 0.1% Tween-20 (TBST) for 1 h. After rinsing in TBST, blots were incubated in anti-BDNF (Epitomics, 2960-1, 1:5,000), anti-D2R (Abcam, ab85367, 1:4,000), or anti-D3R (Santa, sc-9114, 1:500) antibodies with 5% nonfat dried milk at 4°C overnight. Rinsed in TBST again, the blots were then incubated in HRP-conjugated anti-rabbit IgG (1:10,000) for 1 h at room temperature. After several rinses in TBST, target proteins were visualized by enhanced chemiluminescence (Pierce, Rockford, IL, USA) and analyzed by densitometry using a computer-assisted gel quantification system (TotalLab2.01, Phoretix, UK). Western blot data were obtained as background-subtracted optical densities and normalized to glyceraldehyde-3-phosphate dehydrogenase (GAPDH) expression. Normalized values were converted to percent-of-saline for each gel (Change fold).

### 2.7 Data analyses

Data are presented as mean ± standard error of mean (SEM). For all analyses, assumptions for homogeneity of variance and normal distribution of the datasets were verified using the Levene and Shapiro–Wilk tests, respectively. In case of violation of at least one of these assumptions, datasets were log-transformed. A non-parametric test compared the approaching behaviors between saline- and morphine-treated groups. A K-means cluster analysis was performed to identify the high- and low-approaching animals in the conflict-based test and the high- and low-responding animals during PR sessions (Ansquer et al., 2014). One-way Analysis of Variance (ANOVA) was used to analyze approaching behaviors (scores logarithmically transformed). Two-way repeated-measures ANOVA was used to analyze operant responses in FR1 and PR sessions, as well as the contrafreeloading level, with “time” or “FR” as the within-subject factor and “group” or “treatment” as the between-subject factor. The one-way ANOVA was used to analyze protein expression within the brain regions. Tukey's *post hoc* analyses were performed to reveal group differences further. Linear and non-linear correlation/regression analyses were performed between protein expression and appetitive or operant behaviors and among the operant responses on different testing days. Significance level was set at 0.05. All statistical analyses were performed using Statistical Package for the Social Sciences (SPSS) version 25.0 (IBM, Armonk, NY).

## 3 Results

### 3.1 Characterization of high- and low-approaching rats based on the conflict-based appetitive behaviors

A non-parametric test (Mann–Whitney U test) performed on the behavioral scores showed that the morphine-treated group did not differ from the saline-treated group in approaching behaviors (*p* = 0.25; Figure 2B). In view of the large interindividual differences in approaching behaviors, a cluster analysis was performed on the scores that rats acquired in the conflict task to confirm the existence of two subpopulations of rats: high-approaching rats (H, *n* = 8) and low-approaching rats (L, *n* = 16; Figure 2B). Approximately 87% or 7 out of 8 high-approaching rats came from the MOR group, showing that individuals were three times more likely to display perseverative reward-seeking behavior when they had been exposed to morphine than if they had been exposed to saline [Baysian probability: P(H/MOR) = P(MOR/H or 7/8) × P(H or 8/24)/P(MOR or 16/24) = 0.43; while P(H/SAL) = 0.12]. Since only one saline-treated rat was identified as H rat, it was not considered for the following analyses. The log-transformed data [Log(Score)] successfully passed the tests of homogeneity of variance and normal distribution. The morphine-treated H rats displayed significantly more approaching behaviors than either morphine-treated L rats or saline-treated L rats [i.e., SAL group in figure; *F*_(2, 20)_ = 24.72, *p* < 0.0001; MOR-H vs. MOR-L: *p* < 0.0001, MOR-H vs. SAL: *p* < 0.0001; Figure 2C].

### 3.2 Approaching behaviors were associated with D2R within the ventral striatum and D3R within the mPFC in morphine-exposed rats

**Figure 3:**
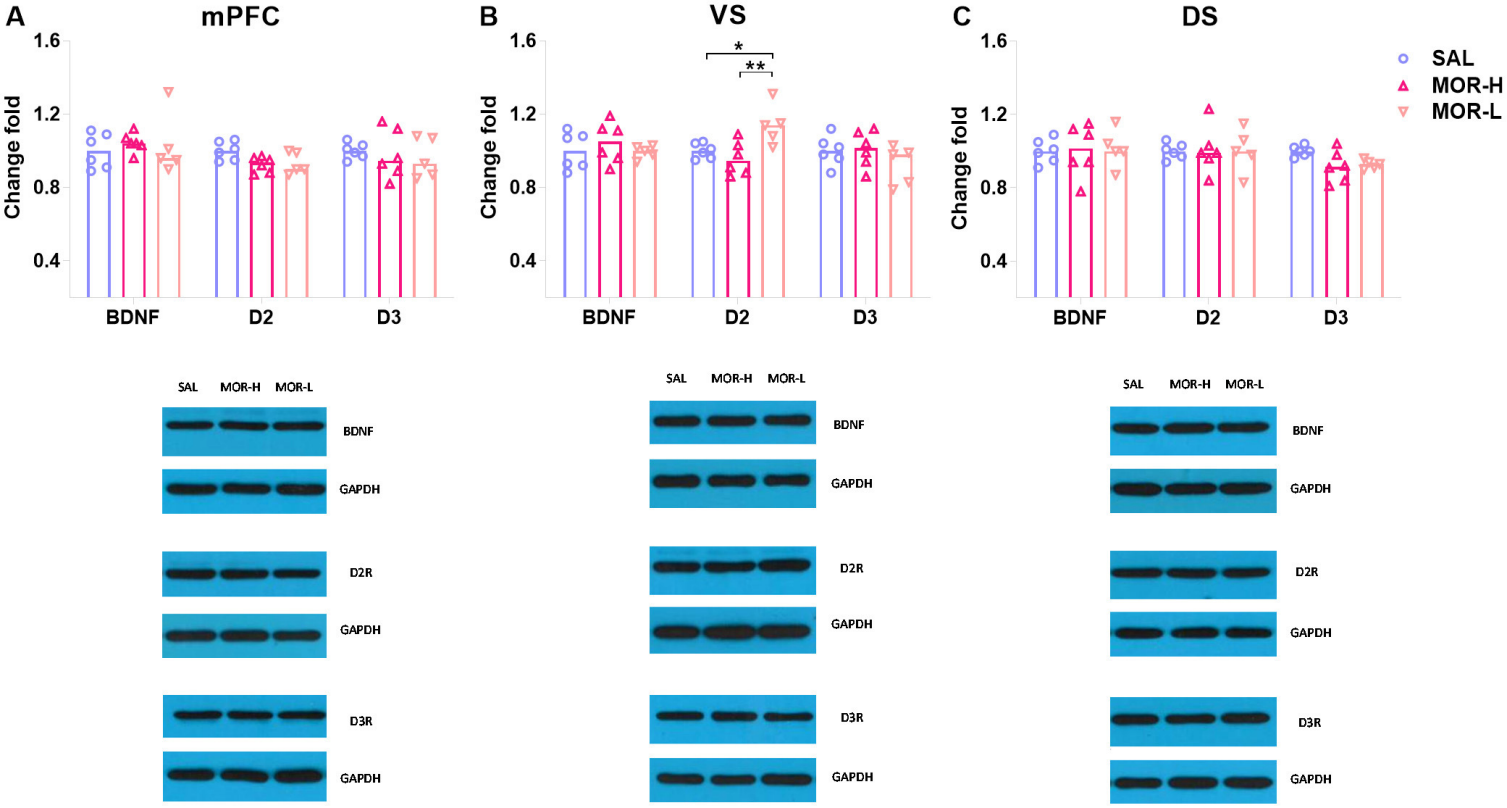
Expressions of D2R, D3R, and BDNF in the mPFC **(A)**, ventral striatum (VS) **(B)**, and dorsal striatum (DS) **(C)** after the conflict-based test. Protein expression was examined 30 min after the test. Data are expressed as mean ± SEM of protein expression percentage vs. saline (Change fold). SAL-L, *n* = 6; MOR-H, *n* = 6; MOR-L, *n* = 5. SAL, saline; MOR, morphine. H, high-approaching; L, low-approaching. *p < 0.05 and **p < 0.01.

Seventeen male rats from SAL, MOR-L, and MOR-H groups (*n* = 6, 5, and 6, respectively) were randomly selected for Western blotting test for D2R, D3R, and BDNF expressions in the mPFC, ventral striatum (VS), and dorsal striatum (DS). The ANOVA showed a significant difference in D2R levels in the mPFC among groups [*F*_(2, 14)_ = 4.01, *p* = 0.042], but *post hoc* analyses only revealed the differences with a trend toward significance between saline- and morphine-treated groups (SAL vs. MOR-L: *p* = 0.1, SAL vs. MOR-H: *p* = 0.05; Figure 3A). The VS D2R level of MOR-H rats was found to be lower than that of MOR-L rats, and the MOR-L and SAL group also significantly differed [*F*_(2, 14)_ = 6.80, *p* < 0.01; MOR-L vs. SAL: *p* = 0.037, MOR-L vs. MOR-H: *p* < 0.01, MOR-H vs. SAL: *p* > 0.7; Figure 3B]. There were significant group differences in D3R level in the DS [*F*_(2, 14)_ = 3.94, *p* = 0.044], but lack of significant *post hoc* comparisons despite a trend toward significance (MOR-L vs. SAL: *p* = 0.12, MOR-H vs. SAL: *p* = 0.05, MOR-L vs. MOR-H: *p* > 0.9; Figure 3C). The mPFC, VS, or DS BDNF level, VS D3R level, or DS D2R level did not show any change following morphine exposure (Statistics were not shown). Thus, there was no significant difference between the groups in any protein measured in the mPFC and DS. The only finding was a significant increase in D2R level in the VS of MOR-L rats.

**Figure 4:**
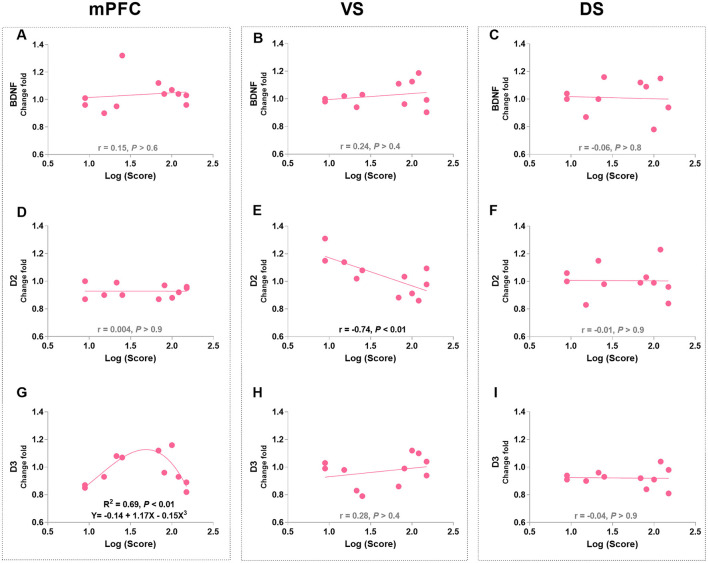
The associations of BDNF, D2R, and D3R in the mPFC, ventral striatum (VS), and dorsal striatum (DS) with approaching behaviors in morphine-treated rats. **(A–C)** Show the correlation relationship between BDNF and behaviors in mPFC, VS and DS, respectively. **(D–F)** Show the correlation between D2R and behaviors in mPFC, VS and DS, respectively. **(G–I)** Show the relationship between D3R and behaviors in mPFC, VS and DS, respectively. Protein expression data are expressed as a percentage vs. saline (Change fold). Behavioral data are represented as logarithmic scores for approaching behavior in the conflict-based test, *N* = 11.

Pearson's correlation analysis was used to identify the linear relationships between protein levels and appetitive behaviors in morphine-treated rats. The VS D2R levels were negatively correlated with approaching behaviors (*r* = −0.74, *p* < 0.01; Figure 4E), while no correlation was found between other protein levels and approaching behaviors (Figure 4). Notably, the level of mPFC D3R was found to be significantly related to approaching behaviors according to a non-linear, third order polynomial relationship {mPFC D3R = −0.14 + 1.17 × Log (Score) - 0.15 × [Log (Score)]^3^, *R*^2^ = 0.69, *p* < 0.01; Figure 4G}. We also merged the groups treated with saline and morphine to examine the general relationship between protein levels in three regions and approaching behaviors. No significant correlation was found between protein level and approaching behavior (statistics and figures were not shown).

### 3.3 Characterization of high- and low-responding rats based on the PR performance

**Figure 5:**
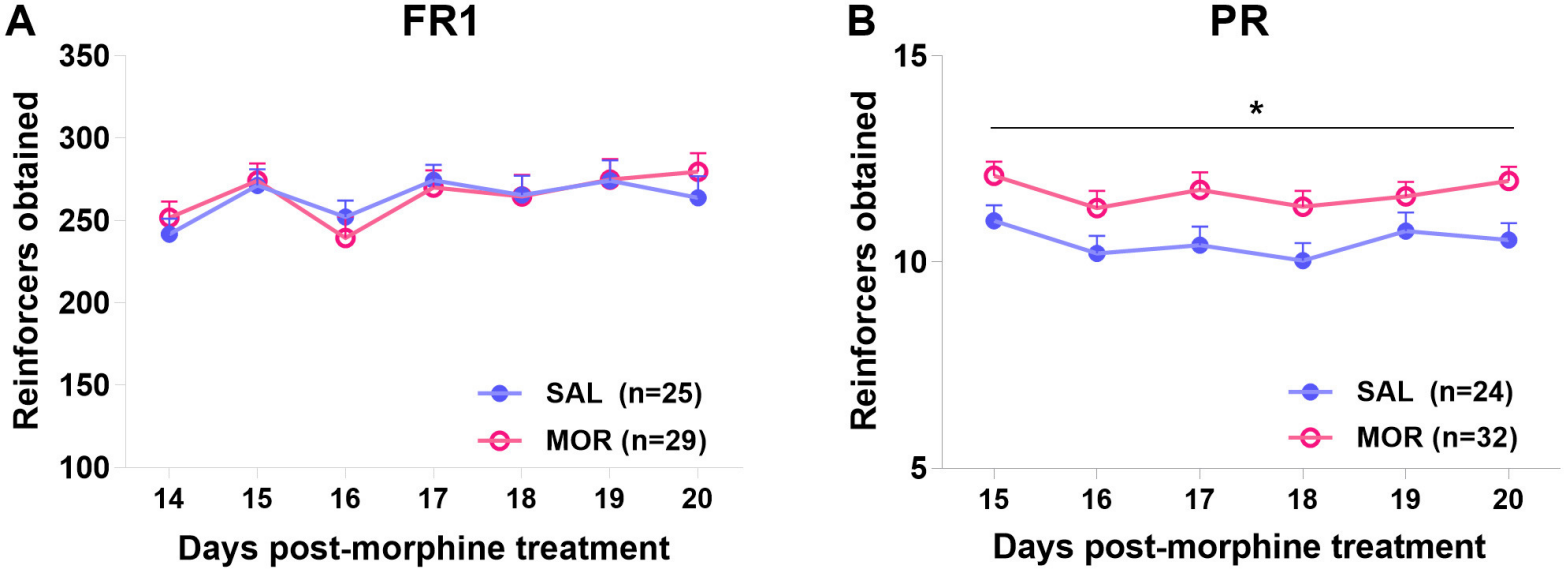
The operant responding under fixed ratio 1 (FR1) **(A)** or progressive ratio (PR) **(B)** procedure after protracted abstinence from morphine. Values are mean ± standard error of mean (SEM) of reinforcers obtained (number of reinforcements) in each session. Rats were treated with saline (SAL) or morphine (MOR). *p < 0.05 (group effect).

Under a PR procedure, the morphine-treated rats exhibited significantly higher responding for 15% sucrose solution than the saline-treated rats across 6 daily sessions [Effect of treatment: *F*_(1, 54)_ = 5.96, *p* = 0.018; Effect of time: *F*_(5, 270)_ = 3.76, *p* < 0.01; Interaction: *F*_(5, 270)_ = 0.42, *p* > 0.8; Figure 5B], while the level of responding under FR1 did not differ between two treatment groups [Effect of treatment: *F*_(1, 52)_ = 0.01, *p* > 0.9; Effect of time: *F*_(6, 312)_ = 9.884, *p* < 0.001; Interaction: *F*_(6, 312)_ = 1.37, *p* > 0.2; Figure 5A].

**Figure 6:**
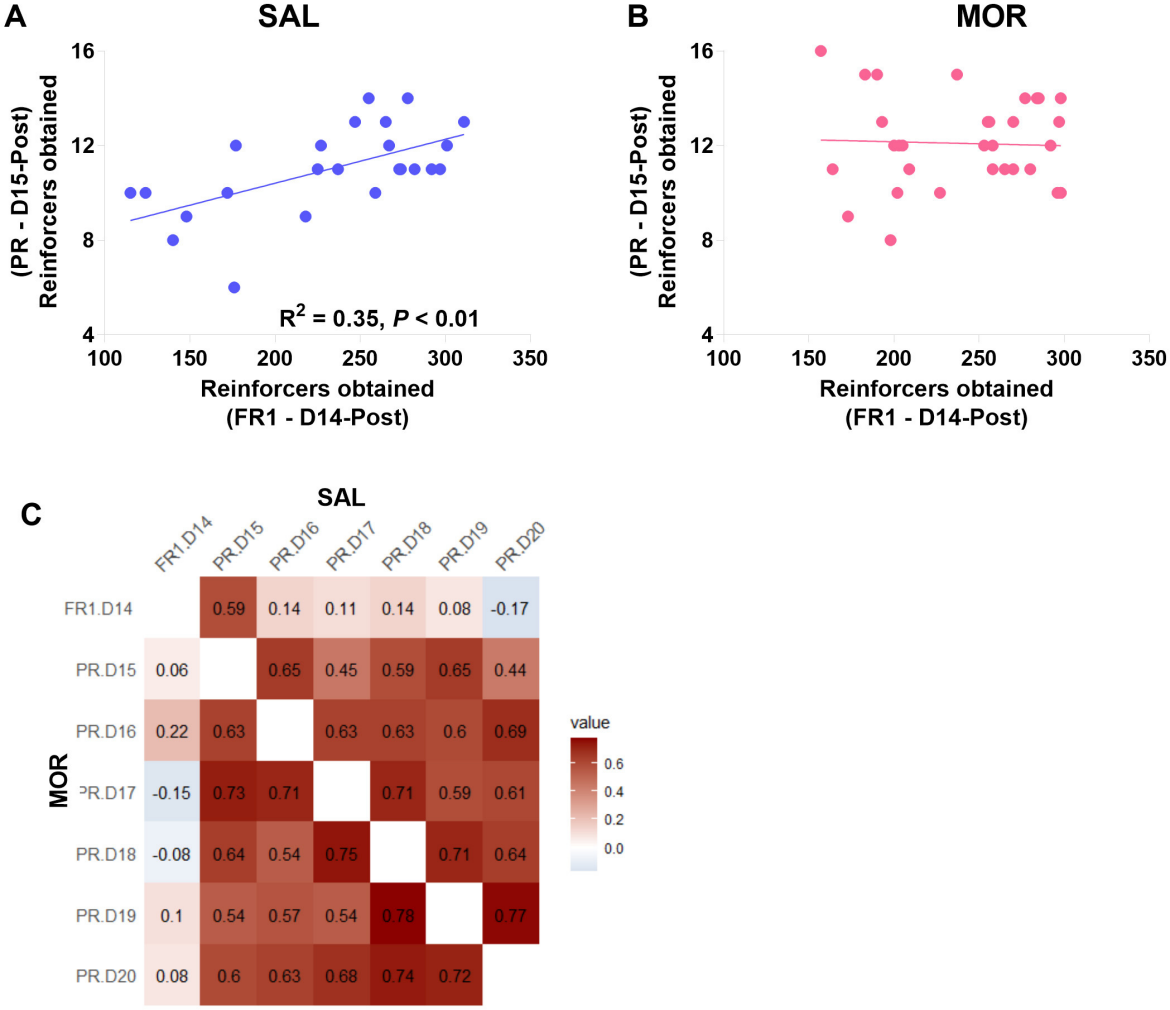
The relations between responding behaviors in the FR1 and PR sessions. The relation of responding behavior under FR1 on day 14 post-morphine treatment (D14-Post) to the responding behavior under PR on day 15 post-morphine treatment (D15-Post) was displayed in this figure **(A, B)**. Data are expressed as reinforcers obtained (number of reinforcements). **(C)** shows the correlation relationship (r) between the responding behaviors in the sessions from day 14 to 20 post-morphine treatment (D14–D20), including the upper right (SAL) and the lower left (MOR) matrices. SAL, saline-treated group, *n* = 24; MOR, morphine-treated group, *n* = 32.

Moreover, in the saline-treated group, responses under the last FR1 (Day 14) were significantly linearly related to responses in the first PR session (day 15; *R*^2^ = 0.35, *p* < 0.01; Figure 6A). At the same time, this relationship was not found in morphine-treated rats (*R*^2^ = 0.001, *p* > 0.80; Figure 6B). Then, responses under the last FR1 were no longer related to responses in any of the previous five PR sessions (day 16–20; Figure 6C).

**Figure 7:**
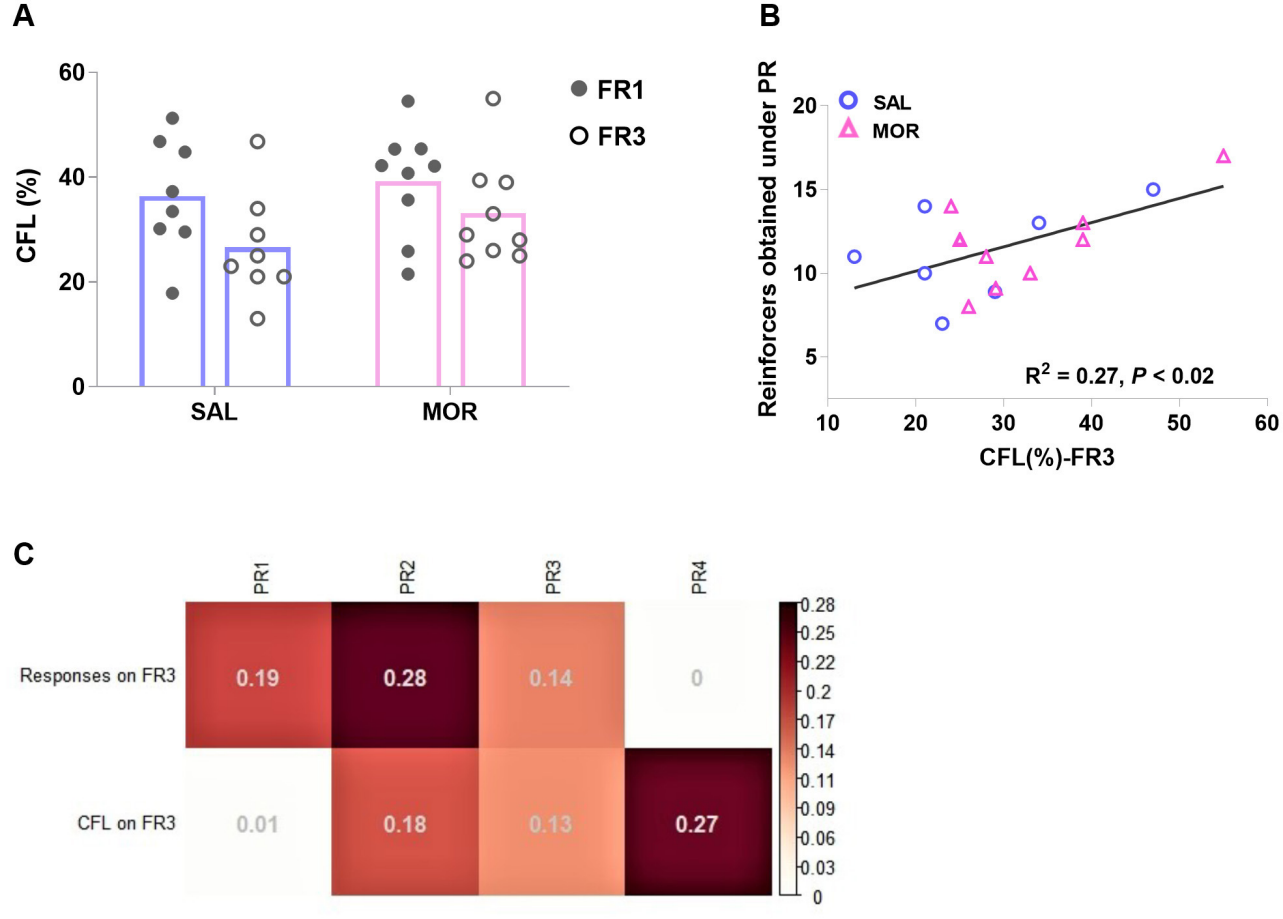
The performance in a contrafreeloading test in which rats had access to a sucrose reward freely or upon instrumental responding concurrently. Contrafreeloading (CFL) level was expressed as the percentage of the fraction of total fluid intake gained instrumentally [CFL(%)]. The CFL level under FR1 or FR3 was displayed by rats after long-term cessation of saline or morphine treatment **(A)**. The CFL level on FR3 is significantly related to the following responding behavior in the fourth PR session **(B)**. **(C)** demonstrates the linear relations of responses on FR3 and CFL level on FR3 to the following responses in four PR sessions (PR1, PR2, PR3, and PR4) (*R*^2^). SAL, saline, *n* = 8; MOR, morphine, *n* = 9. *p < 0.05, CFL%-FR1 vs. CFL%-FR3.

In a contrafreeloading test, rats were chosen between freely available sucrose and delivered sucrose via responding under FR. The CFL level (percentage of delivered sucrose) significantly declined after the workload increased from FR1 to FR3 [Effect of FR: *F*_(1, 15)_ = 6.91, *p* < 0.02; Effect of treatment: *F*_(1, 15)_ = 1.38, *p* > 0.2; no interaction: *F*_(1, 15)_ = 0.37, *p* > 0.5; Figure 7A]. When two variables, responses on FR3 and the CFL on FR3, were involved into the linear regression analyses to predict the following responses over four PR sessions. The responses on FR3 were significantly related to the responses in the first and second, but not third or fourth PR sessions ($R_{first}^{2}$ = 0.19, *p* = 0.047 and $R_{second}^{2}$ = 0.28, *p* = 0.018; Figure 7C). However, the CFL on FR3 was found to be significantly related to the responses in the fourth, but not first, second, or third PR session ($R_{fourth}^{2}$ = 0.27, *p* = 0.018; Figure 7B).

**Figure 8:**
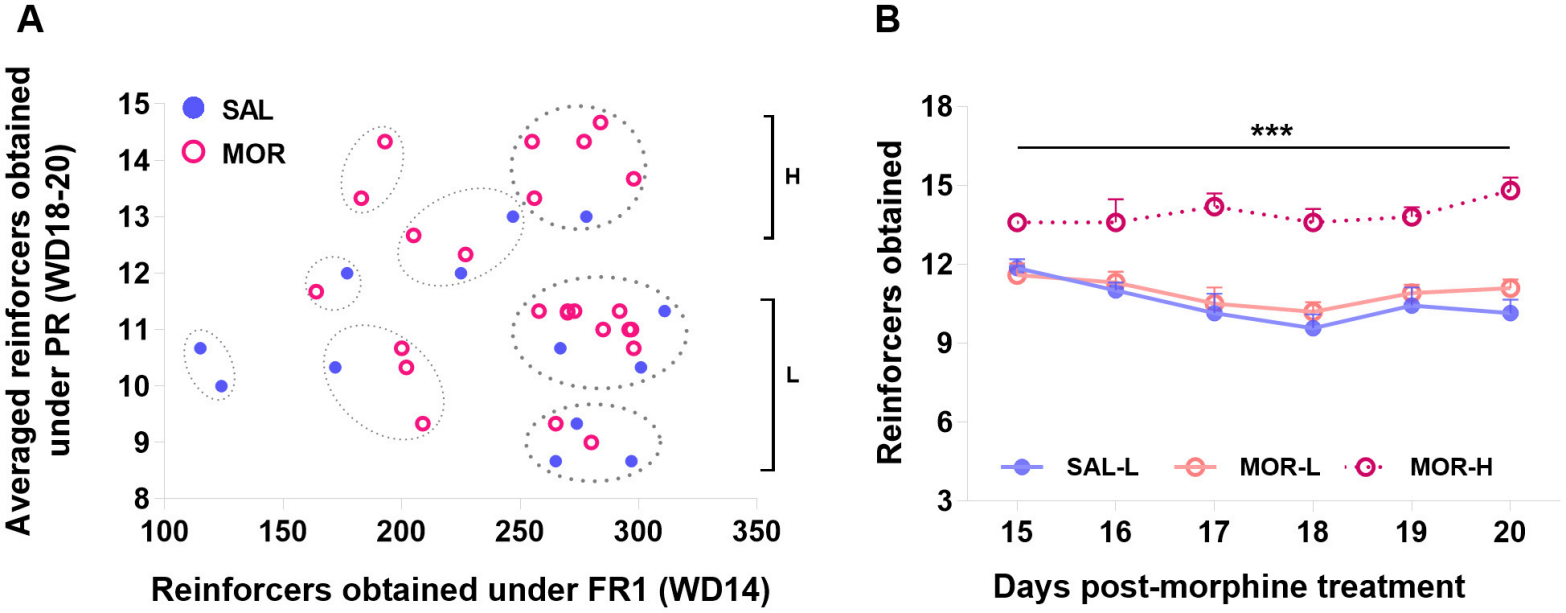
Characterization of the high- and low-responding rats based on the PR performance. Eight subpopulations of rats were identified by a K-means cluster analysis based on the FR1 responding and the averaged responding in the last three PR sessions. The subpopulations in which FR1 responding was above 250 were designated as high-responding (H) or low-responding (L) rats according to their PR responding **(A)**. The responding behaviors of H and L subpopulations during six PR sessions were compared (SAL-L, *n* = 7; SAL-H, *n* = 1; MOR-L, *n* = 10; MOR-H, *n* = 5) **(B)**. Data are expressed as data points of each rat (a) or mean ± SEM **(B)**. SAL, saline; MOR, morphine. D14-Post, day 14 post-morphine treatment; D18~20-Post, day 18~20 post-morphine treatment. ***p < 0.001.

Also considering the individual difference in PR responding, a cluster analysis was performed on two dimensions: FR1 responses (day 14 post-morphine treatment) and the averaged responses over the last three PR sessions (day 18–20 post-morphine treatment), confirming eight distinct subpopulations. About 62% of rats (*n* = 23) that displayed responses above 250 (reinforcers obtained) under FR1 belonged to 3 subpopulations. The rest of the rats (*n* = 14) that displayed responses below 250 were spread in 5 subpopulations and laid aside in this study. According to the responding level under PR, the 23 rats were further classified as high-responding rats (H, *n* = 6) and low-responding rats (L, *n* = 17; Figure 8A). Approximately 83% or 5 out of 6 high-responding rats came from the MOR group, showing that individuals were nearly three times more likely to display high-responding behavior when they had been exposed to morphine than if they had been exposed to saline (Baysian probability: P(H/MOR) = P(MOR/H or 5/6) × P(H or 6/23)/P(MOR or 15/23) = 0.33; while P(H/SAL) = 0.12). Since only one saline-treated rat was identified as H rat, it was discarded from the following analyses. The MOR-H rats earned significantly more reinforcers than both MOR-L and SAL-L rats (i.e., SAL group in figures) over six PR sessions [Group effect: *F*_(2, 19)_ = 30.24, *P* < 0.0001; Time effect: *F*_(5, 95)_ = 2.61, *P* < 0.05; No interaction; *post hoc*: MOR-H vs. MOR-L: *P* < 0.0001, MOR-H vs. SAL: *P* < 0.0001, MOR-L vs. SAL: *P* > 0.7; Figure 8B].

### 3.4 PR responding behaviors were associated with D3R within the dorsal striatum, D3R and BDNF within the mPFC in morphine-exposed rats

**Figure 9:**
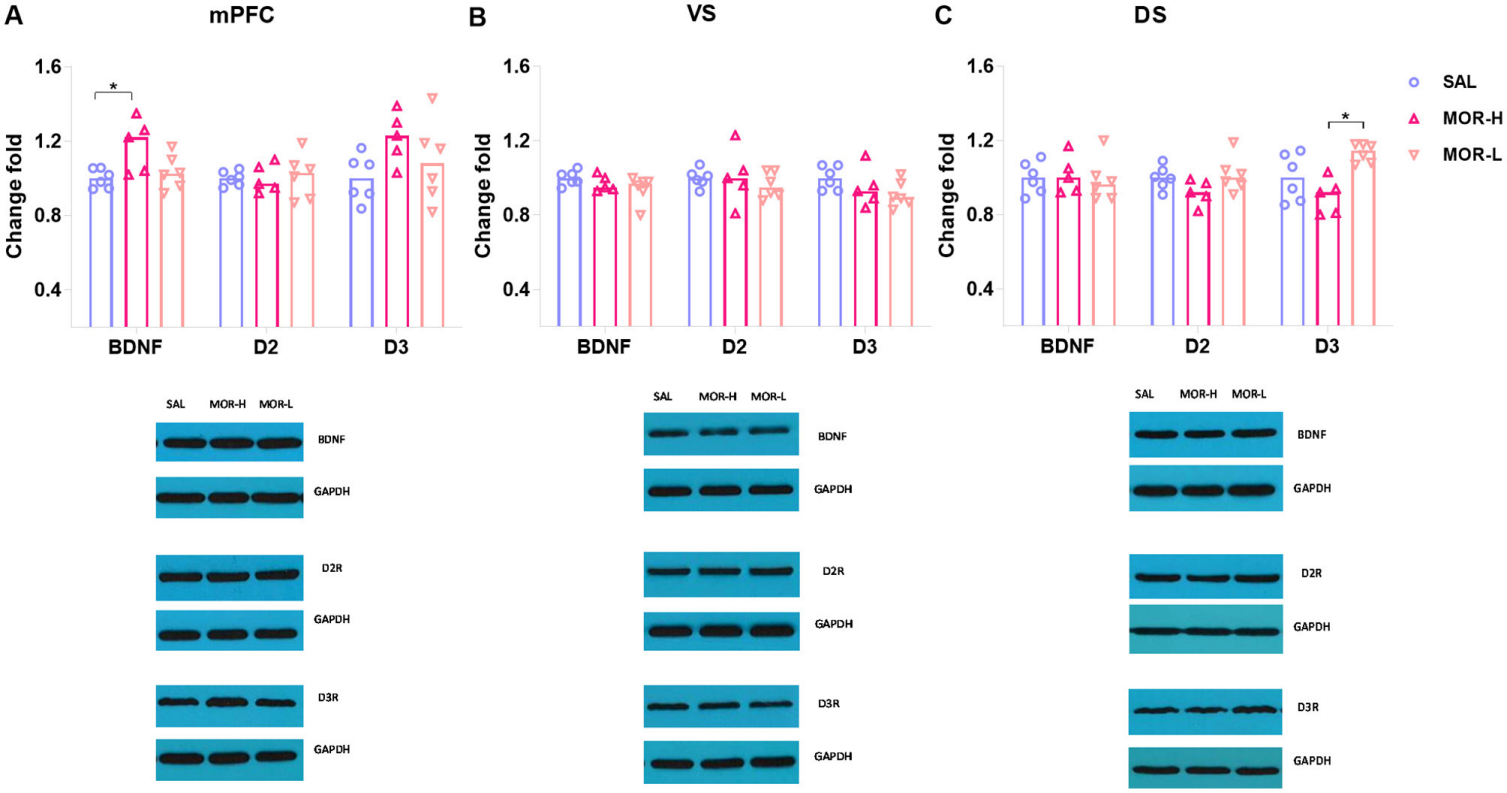
Expressions of D2R, D3R, and BDNF in the mPFC **(A)**, ventral striatum (VS) **(B)**, and dorsal striatum (DS) **(C)** after the operant task. Protein expression was examined 30 min after the last (sixth) PR session. Data are expressed as mean ± SEM of protein expression percentage vs. saline (Change fold). SAL-L, *n* = 6; MOR-H, *n* = 5; MOR-L, *n* = 6. SAL, saline; MOR, morphine. H, high-responding; L, low-responding. *p < 0.05.

Twelve rats from the L subpopulation (MOR-L: *n* = 6; SAL: *n* = 6) and five rats from the H subpopulation (MOR-H) were randomly selected for the Western blotting test. The MOR-H rats had the increased BDNF expression in the mPFC [BDNF: *F*_(2, 14)_ = 4.88, *P* = 0.025, MOR-H vs. SAL: *P* = 0.025, MOR-L vs. SAL: *P* > 0.8, MOR-H vs. MOR-L: *P* = 0.07; Figure 9A]. There was a remarkable difference in D3R level within the DS between MOR-H and MOR-L groups, and the difference with a trend toward significance was found when comparing the MOR-L group to the SAL group [*F*_(2, 14)_ = 8.13, *P* < 0.01, MOR-L vs. MOR-H: *P* < 0.01, MOR-L vs. SAL: *P* = 0.08, MOR-H vs. SAL: *P* > 0.2; Figure 9C]. The other protein expressions did not show significant difference among three groups (*Ps* > 0.05; e.g., Figure 9B).

**Figure 10:**
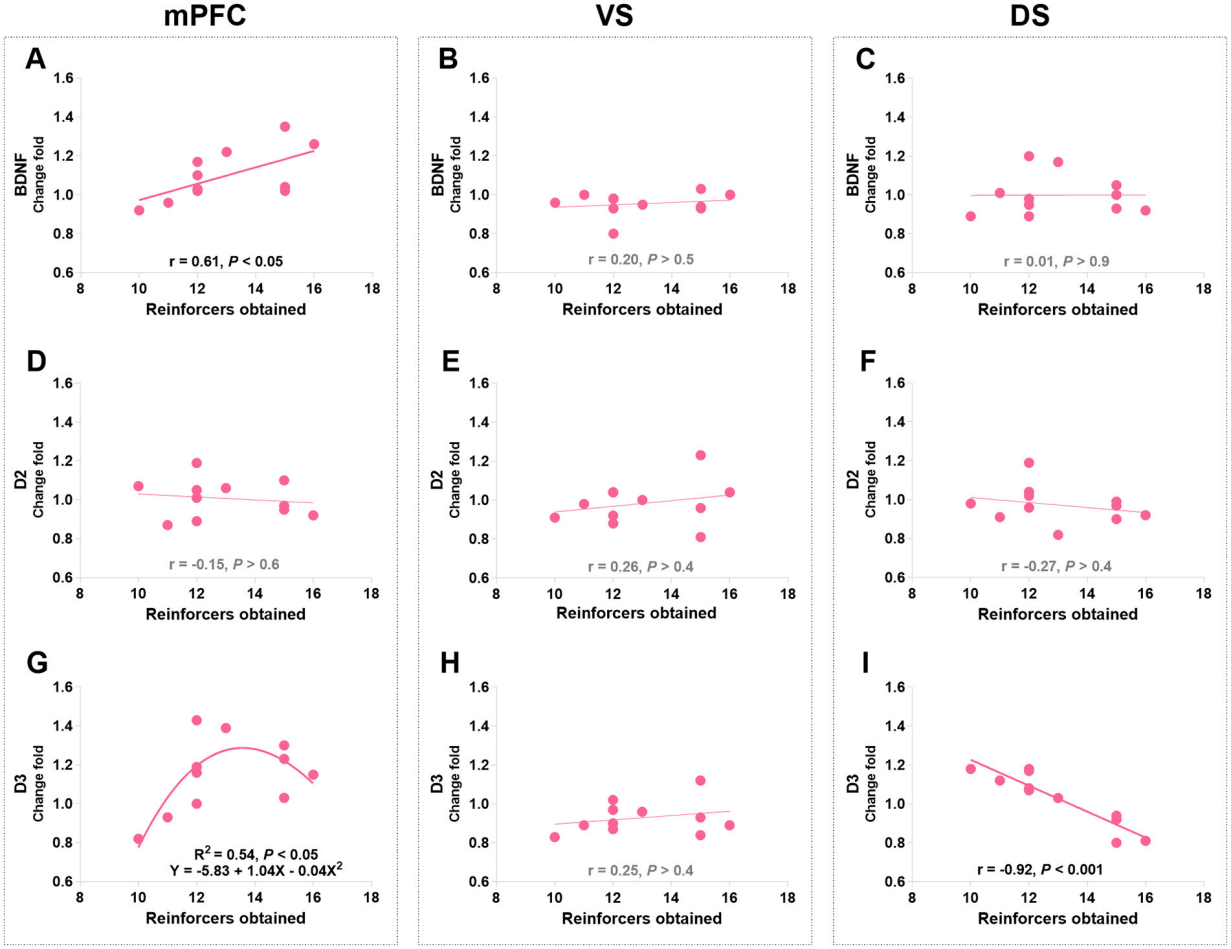
The associations of BDNF, D2R, and D3R in the mPFC, ventral striatum (VS), and dorsal striatum (DS) with the responding behaviors in morphine-treated rats. **(A–C)** Show the correlation relationship between BDNF and behaviors in mPFC, VS and DS, respectively. **(D–F)** Show the correlation between D2R and behaviors in mPFC, VS and DS, respectively. **(G–I)** Show the relationship between D3R and behaviors in mPFC, VS and DS, respectively. Protein expression data are expressed as a percentage vs. saline (Change fold). Behavioral data are represented as reinforcers obtained (number of reinforcements) in the last (sixth) PR session. *N* = 11.

**Figure 11:**
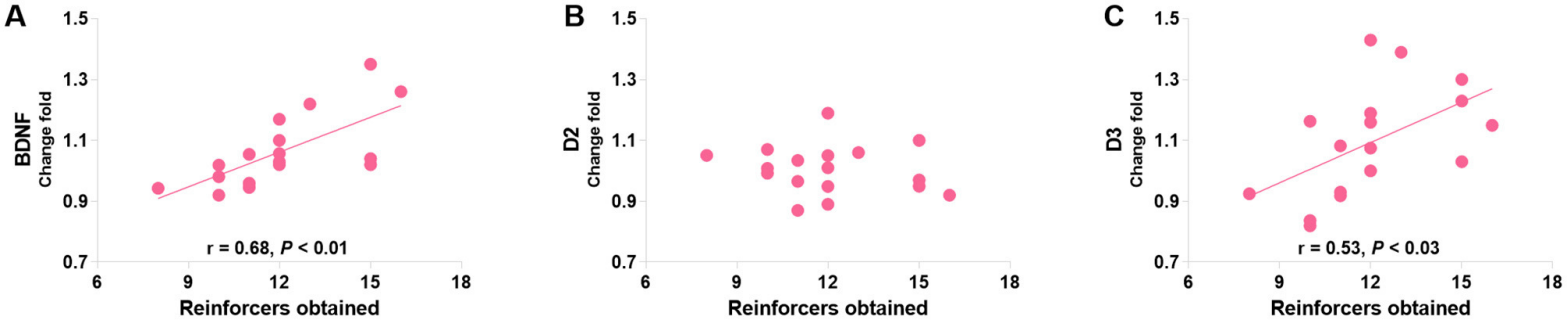
The associations of BDNF **(A)**, D2R **(B)**, and D3R **(C)** in the mPFC with the responding behaviors in a group consisting of saline- and morphine-treated rats. Protein expression data are expressed as percentage vs. saline (Change fold). Behavioral data are represented as reinforcers obtained (number of reinforcements) in the last (sixth) PR session. *N* = 17.

Pearson's correlation analyses were used to identify the relationships between protein levels and responding behaviors in the last PR session (day 20 post-morphine treatment) in morphine-treated rats. The only significant findings are that PR performance positively correlated with the mPFC BDNF level (*r* = 0.61, *P* = 0.046; Figure 10A) and negatively correlated with the DS D3R level (*r* = −0.92, *P* < 0.001; Figure 10I). Consistent with the finding in the conflict task, the mPFC D3R level was related to the PR performance according to a non-linear, second order polynomial relationship [mPFC D3R = −5.83 + 1.04 × reinforcers obtained - 0.04 × (reinforcers obtained)^2^, *R*^2^ = 0.54, *p* = 0.044; Figure 10G]. No significant differences were found in other protein expressions (Figures 10B–F, H). We also merged the groups treated with saline and morphine to examine the general relationships of protein levels in three regions to responding behaviors. The significant findings are that responding behavior in the last PR session is positively correlated with BDNF and D3R expression in the mPFC (*r* = 0.68, *p* < 0.01; *r* = 0.53, *p* = 0.029; Figures 11A, C).

## 4 Discussion

In our previous studies, we observed that exposure to morphine resulted in increased approaching behaviors toward sexual stimuli in the presence of a dangerous obstacle in a conflict task, as well as higher engagement in pursuing sucrose under a progressive ratio (PR) procedure. These high-cost behaviors related to perseveration persisted long after withdrawal from morphine and exhibited significant individual differences (Bai et al., 2014, 2017; Li et al., 2017). In this study, we further classified the rats into subpopulations based on their performance in these two tasks. We found that rats treated with morphine were more likely to develop high-performance behaviors, i.e., high-approaching and high-responding.

Using fixed ratio 1 (FR1) and PR procedures allowed us to investigate different motivational constructs (Markou et al., 1993; Brennan et al., 2001). Consistent with previous research, we found that the significant difference between the two treatment groups was observed in responding behaviors under the PR procedure but not the FR1 procedure (Figure 5). Additionally, we observed no overall relationship between FR1 responding and subsequent PR responding (Figure 6C), except for a positive relationship between FR1 responding and responding in the first PR session in saline-treated rats (Figure 6A). This suggests that drug-naive rats relied on their experiences from FR1 sessions to guide their behaviors under the new contingency of reinforcement (PR), at least partially based on their preferences and desires for sucrose. However, this relationship was absent in morphine-treated rats (Figure 6B), indicating that their motivation toward sucrose was distorted by the uncertainty introduced by the new contingency in the initial PR session. Furthermore, we found that PR responses may reflect more than just motivation. By conducting a contrafreeloading (CFL) task, we discovered that the unnecessary/perseverative responding behaviors (the CFL level on FR3) explained a portion of responding during the fourth PR session. And the responding in the first two PR sessions was predicted to some extent by prior responding on the CFL task (responding on FR3), but not by the CFL level itself (Figures 7B, C). This suggests that repeated training under the PR procedure may facilitate the development of perseverative responding behaviors, which may not only reflect the reinforcing efficacy of the reward and motivation but also perseverative behaviors. Moreover, we observed that morphine-treated rats were more likely to develop high-responding behaviors after repeated PR sessions than saline-treated rats (Figure 8A). Taken together with the findings from the conflict task, it is suggested that a history of opioid exposure enhances perseverative reward-seeking behaviors, indicating impaired inhibitory control in some individuals.

Previous studies have consistently reported lower availability of dopamine D2 receptors (D2Rs) in the striatum of individuals dependent on cocaine, opiates, and other substances during abstinence (Martinez et al., 2004; Volkow et al., 2004; Martinez et al., 2005). Similarly, rodents with a history of repeated morphine administration or chronic heroin/cocaine self-administration also exhibit reduced D2Rs in the striatum (Turchan et al., 1997; Spangler et al., 2003; Conrad et al., 2010; Tacelosky et al., 2015). However, in this study, we did not observe a reduction in D2R expression in either striatum region. This may be attributed to the prolonged withdrawal period, as previous studies have shown that the decrease in D2R mRNA and availability induced by morphine or cocaine administration returns to normal levels with an extended withdrawal period (Georges et al., 1999; Nader et al., 2006). Furthermore, there are individual variations in the rate of recovery of D2R availability, with some individuals never fully recovering D2R availability even after 12 months of abstinence (Nader et al., 2006). Although we did not examine the longitudinal changes in D2R expression since the cessation of morphine administration, we did observe individual differences in the level of D2R in the ventral striatum (VS), which were negatively correlated with approaching behaviors in morphine-treated rats (Figure 4E). In addition, morphine-treated low-approaching rats exhibited increased D2R expression in the VS compared to drug-naive rats and morphine-treated high-approaching rats (Figure 3B). Low striatal dopamine D2Rs in humans and rodents, whether prior to or after drug exposure, represent a risky marker for trait-like waiting impulsivity (Dalley et al., 2007; Caprioli et al., 2013; Simon et al., 2013; Barlow et al., 2018), dependence vulnerability (Volkow et al., 1999, 2002; Dalley et al., 2007; Belin et al., 2008) and enhanced impulsivity after drug abuse (Dawe and Loxton, 2004; Lee et al., 2009; Ballard et al., 2015). On the contrary, a high level of D2Rs may be protective against alcoholism, as a few studies have discovered that the adenoviral vector-mediated overexpression of D2R in the VS can reduce alcohol preference and intake in rats (Thanos et al., 2001, 2004). Moreover, the unaffected members of alcoholic families (social drinkers) are found to have higher D2R availability in the striatum than the family-history-negative social drinkers (Volkow et al., 2006; Alvanzo et al., 2015). Consistent with these studies, the findings in our study provide additional support for the notion that a higher level of D2R in the VS may be protective for individuals, allowing them to maintain standard control over their reward-seeking behaviors after a history of opioid exposure.

It is worth noting that the alteration of D2R expression was not consistent across all morphine-treated groups, varying depending on the behavioral tasks and brain regions examined. This is also true for D3R and brain-derived neurotrophic factor (BDNF) expressions. D3R, which has a more restricted distribution pattern compared to D2R, is preferentially expressed in the mesolimbic systems that play a crucial role in reward and motivation (Bouthenet et al., 1991; Levesque et al., 1992; Landwehrmeyer et al., 1993a,b; Murray et al., 1994; Gurevich and Joyce, 1999; Clarkson et al., 2017). Many studies have reported elevated D3R levels in the VS of cocaine victims and animals administered with cocaine (Staley and Mash, 1996; Segal et al., 1997; Mash and Staley, 1999; Le Foll et al., 2002; Neisewander et al., 2004; Le Foll et al., 2005; Conrad et al., 2010; Collins et al., 2011). Similarly, repeated administration of morphine and long-term alcohol intake have been shown to increase D3R mRNA in the dorsal striatum (DS) of rats (Spangler et al., 2003; Jeanblanc et al., 2006; Vengeliene et al., 2006). In contrast, in our study, we did not observe significant changes in overall D3R expression after morphine exposure and behavioral tasks. Nevertheless, we did find increased D3R expression in the DS of the morphine-treated low-approaching group compared to the morphine-treated high-approaching group after continuous PR training (Figure 9C). Additionally, there was a strong negative correlation between DS D3R levels and PR responses (Figure 10I), suggesting that the interaction between PR training and morphine exposure may have led to differential expressions of D3R between the high and low subgroups.

Interestingly, we observed a positive correlation between mPFC D3R expression and PR responses in merged groups of saline- and morphine-treated rats (Figure 11C), indicating a general association between D3R and operant behaviors reinforced by sucrose. Previous research has shown that D3R expression highly depends on dopamine neuron activity (Lévesque et al., 1995). Increase in phasic dopamine may invoke D3R regulatory responses, since dopamine receptor agonists (dopamine and quinpirole) induce rapid upregulation of D3R in cell lines (Cox et al., 1995) and 3-h incubation by alcohol induced significant expression of D3Rs *in vitro* (Jeanblanc et al., 2006). Therefore, the linear relationship between mPFC or DS D3R expression and PR responses observed in our study may reflect reactive alterations of D3R following consecutive PR training, during which dopamine release undergoes changes. This aligns with the view that D3R is involved in reinforcement learning, particularly in tasks with high work requirements, such as the PR schedule (Di Ciano et al., 2002; Xi et al., 2005; Ross et al., 2007; Xi and Gardner, 2007; Higley et al., 2011a,b; Song et al., 2012; Chen et al., 2014; Galaj et al., 2013; Galaj et al., 2018).

Furthermore, in morphine-treated rats, we discovered a non-linear relationship (approximate inverted U-shape) between responses in the last PR session and mPFC D3R levels (Figure 10G). Interestingly, the same non-linear relationship was observed in morphine-treated rats during the conflict task (Figure 4G), suggesting that rats displaying more perseverative behaviors did not necessarily have the highest levels of D3R. This finding was further supported by the significantly lower levels of D3R in the DS of morphine-treated high-responding rats compared to morphine-treated low-responding rats (Figure 9C), as well as the strong negative correlation between DS D3R levels and PR responding behaviors (Figure 10I). The role of relatively low D3R expression in these brain regions after morphine exposure remains unclear. Previous studies have suggested that low D3R availability in the brain may be a risk factor, as genetic deletion of D3R in mice leads to increased seeking/perseverative behaviors for sucrose, heroin, and cocaine under PR and extinction procedures (Song et al., 2011, 2012; Zhan et al., 2018). To date, no other studies have reported substance abuse-related alterations in D3R expression in the prefrontal cortex. D3R has been observed in the L5 prefrontal cortex in both primates and rodents (Bouthenet et al., 1991; Lidow et al., 1998). It has been reported to exert inhibitory effects on mesocortical dopamine activity (Gross and Drescher, 2012). Impaired dopamine signaling, possibly via D3R, in the prefrontal regions of the brain can disrupt executive processes and weaken the ability to resist intense urges (Goldstein and Volkow, 2011; Volkow et al., 2016).

BDNF is required for the expression of D3R in the VS, as previous studies have shown that local infusion of BDNF induces D3R expression, whereas BDNF deprivation selectively reduces D3R expression in rats (Guillin et al., 2001). The cortical neuron groups contain high levels of BDNF mRNA (Seroogy et al., 1994; Altar et al., 1997; Conner et al., 1997). Operations that increase dopamine activity, such as exposure to substances of abuse or administration of levodopa in the 6-OHDA-lesioned rat, have been found to induce BDNF expression in the frontal cortex (Guillin et al., 2001; Le Foll et al., 2005). However, very few studies have investigated whether BDNF regulates D3R expression in the prefrontal cortex. Our study observed positive correlations between responding behaviors and both BDNF and D3R in the mPFC after consecutive PR sessions (Figure 11), suggesting a possible regulatory relationship between these two molecules in this region. BDNF may influence dopamine responsiveness by regulating D3R expression in the mPFC and VS, thereby participating in the regulation of behaviors. In the DS, where basal levels of BDNF and D3R expression are low (Nakamura et al., 1996; Diaz et al., 2000), alcohol intake has been shown to increase the levels of BDNF and D3R in rats (Mcgough et al., 2017; Vengeliene et al., 2006). Repeated administration of levodopa in unilaterally 6-OHDA-lesioned rats also triggers dorsal striatal D3R overexpression (Bordet et al., 1997), which is induced by BDNF originating partly from cortical neurons (Guillin et al., 2001). However, in our study, we did not observe parallel alterations in BDNF and D3R expression in the DS, suggesting alternative regulatory mechanisms for D3R beyond BDNF in this region.

In summary, this study employed two high-cost tasks to characterize two phenotypes of perseverative reward-seeking behaviors observed in subpopulations of rats after abstinence from opioids. We also identified behaviorally and regionally specific alterations in D2R and D3R expression in the mPFC and striatum. Our findings provide novel evidence supporting the idea that higher levels of D2R and D3R in the VS and DS, respectively, may represent protective factors for individuals abstinent from opioids, allowing them to maintain control over their reward-seeking behaviors in the face of adversity. The non-linear relationship between mPFC D3R levels and reward-seeking behaviors observed in our study suggests the involvement of D3R in appetitive behaviors and behavioral perseveration. This finding supports a previous study showing that manipulating D3R activity with agonists and antagonists improved compulsive nose-poke behavior in a stop-signal task (Bari and Robbins, 2013). Further research is needed to fully understand the exact relationship between D3R and perseverative behavior and the underlying mechanisms. Identifying molecular markers and neural targets associated with deficits in behavioral control during the drug abstinence period would greatly aid in recognizing and intervening in individuals at risk of relapse.

## Data Availability

The datasets presented in this study can be found in online repositories. The names of the repository/repositories and accession number(s) can be found in the article/Supplementary material.
