# Supplementary material for: Higher levels of D2R and D3R in the frontal–striatal regions are associated with reduced perseverative reward seeking after opioid abstinence
Source: Front Behav Neurosci. 2025 Jun 2;19:1552055. doi: 10.3389/fnbeh.2025.1552055 (PMC12171296; doi:10.3389/fnbeh.2025.1552055)
Supplement: Supplementary file 1 [file Presentation_1.pptx]

## Slide 1
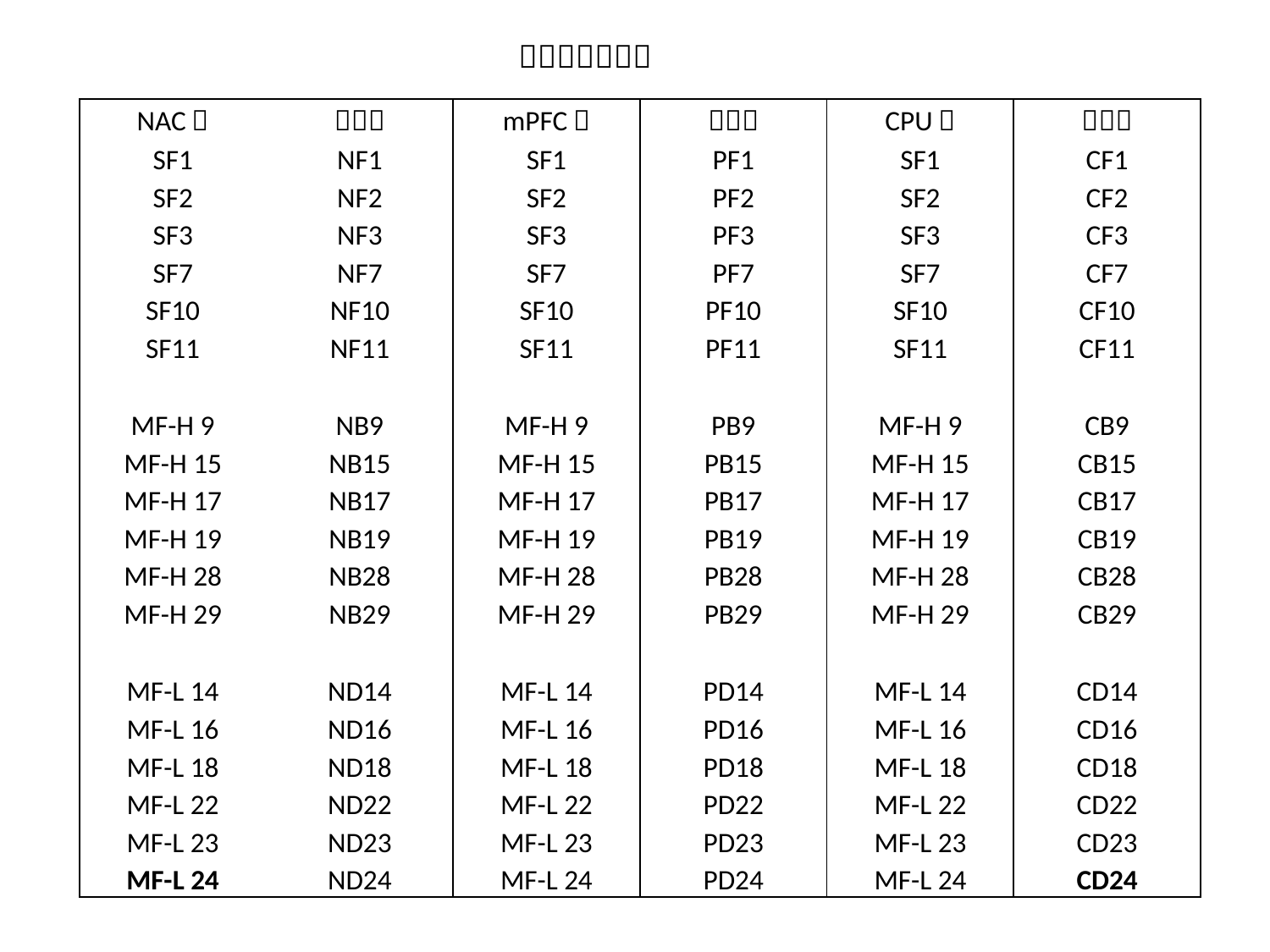

样本新编号对照
| NAC组 | 新编号 | mPFC组 | 新编号 | CPU组 | 新编号 |
| --- | --- | --- | --- | --- | --- |
| SF1 | NF1 | SF1 | PF1 | SF1 | CF1 |
| SF2 | NF2 | SF2 | PF2 | SF2 | CF2 |
| SF3 | NF3 | SF3 | PF3 | SF3 | CF3 |
| SF7 | NF7 | SF7 | PF7 | SF7 | CF7 |
| SF10 | NF10 | SF10 | PF10 | SF10 | CF10 |
| SF11 | NF11 | SF11 | PF11 | SF11 | CF11 |
| | | | | | |
| MF-H 9 | NB9 | MF-H 9 | PB9 | MF-H 9 | CB9 |
| MF-H 15 | NB15 | MF-H 15 | PB15 | MF-H 15 | CB15 |
| MF-H 17 | NB17 | MF-H 17 | PB17 | MF-H 17 | CB17 |
| MF-H 19 | NB19 | MF-H 19 | PB19 | MF-H 19 | CB19 |
| MF-H 28 | NB28 | MF-H 28 | PB28 | MF-H 28 | CB28 |
| MF-H 29 | NB29 | MF-H 29 | PB29 | MF-H 29 | CB29 |
| | | | | | |
| MF-L 14 | ND14 | MF-L 14 | PD14 | MF-L 14 | CD14 |
| MF-L 16 | ND16 | MF-L 16 | PD16 | MF-L 16 | CD16 |
| MF-L 18 | ND18 | MF-L 18 | PD18 | MF-L 18 | CD18 |
| MF-L 22 | ND22 | MF-L 22 | PD22 | MF-L 22 | CD22 |
| MF-L 23 | ND23 | MF-L 23 | PD23 | MF-L 23 | CD23 |
| MF-L 24 | ND24 | MF-L 24 | PD24 | MF-L 24 | CD24 |

## Slide 2
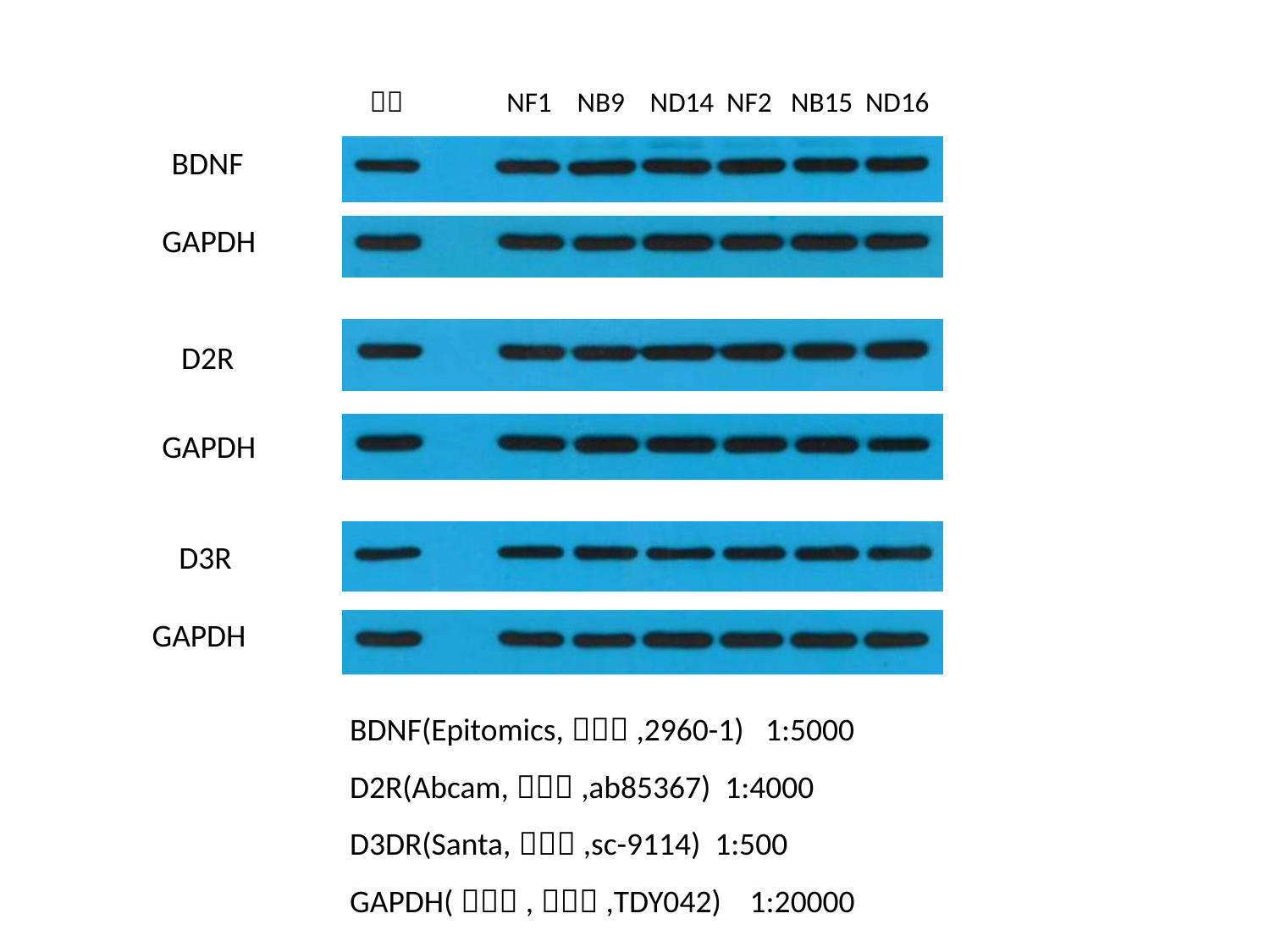

公共 NF1 NB9 ND14 NF2 NB15 ND16
BDNF
GAPDH
D2R
GAPDH
D3R
GAPDH
BDNF(Epitomics,兔单抗,2960-1) 1:5000
D2R(Abcam,兔多抗,ab85367) 1:4000
D3DR(Santa,兔多抗,sc-9114) 1:500
GAPDH(天德悦,鼠单抗,TDY042) 1:20000

## Slide 3
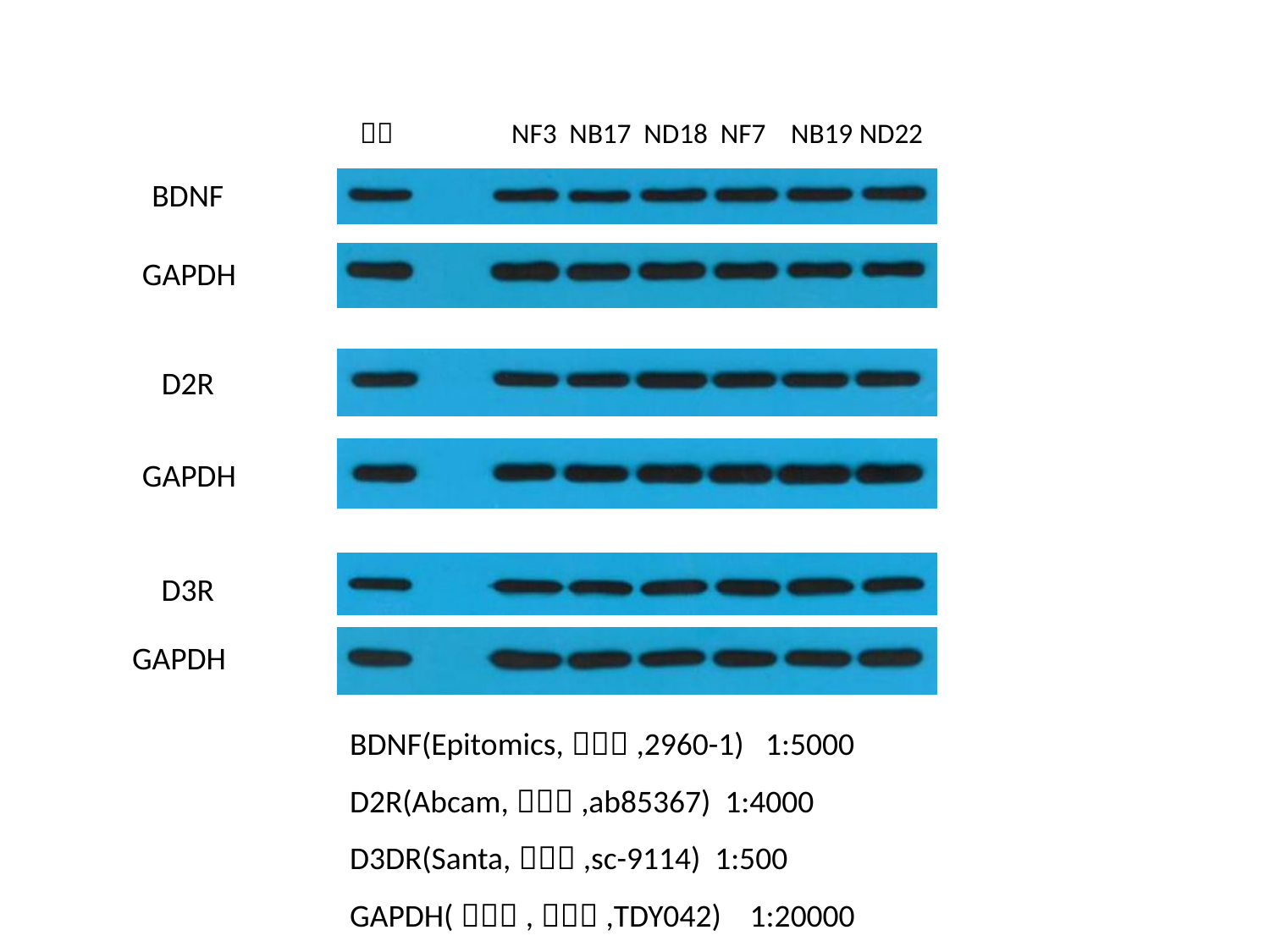

公共 NF3 NB17 ND18 NF7 NB19 ND22
BDNF
GAPDH
D2R
GAPDH
D3R
GAPDH
BDNF(Epitomics,兔单抗,2960-1) 1:5000
D2R(Abcam,兔多抗,ab85367) 1:4000
D3DR(Santa,兔多抗,sc-9114) 1:500
GAPDH(天德悦,鼠单抗,TDY042) 1:20000

## Slide 4
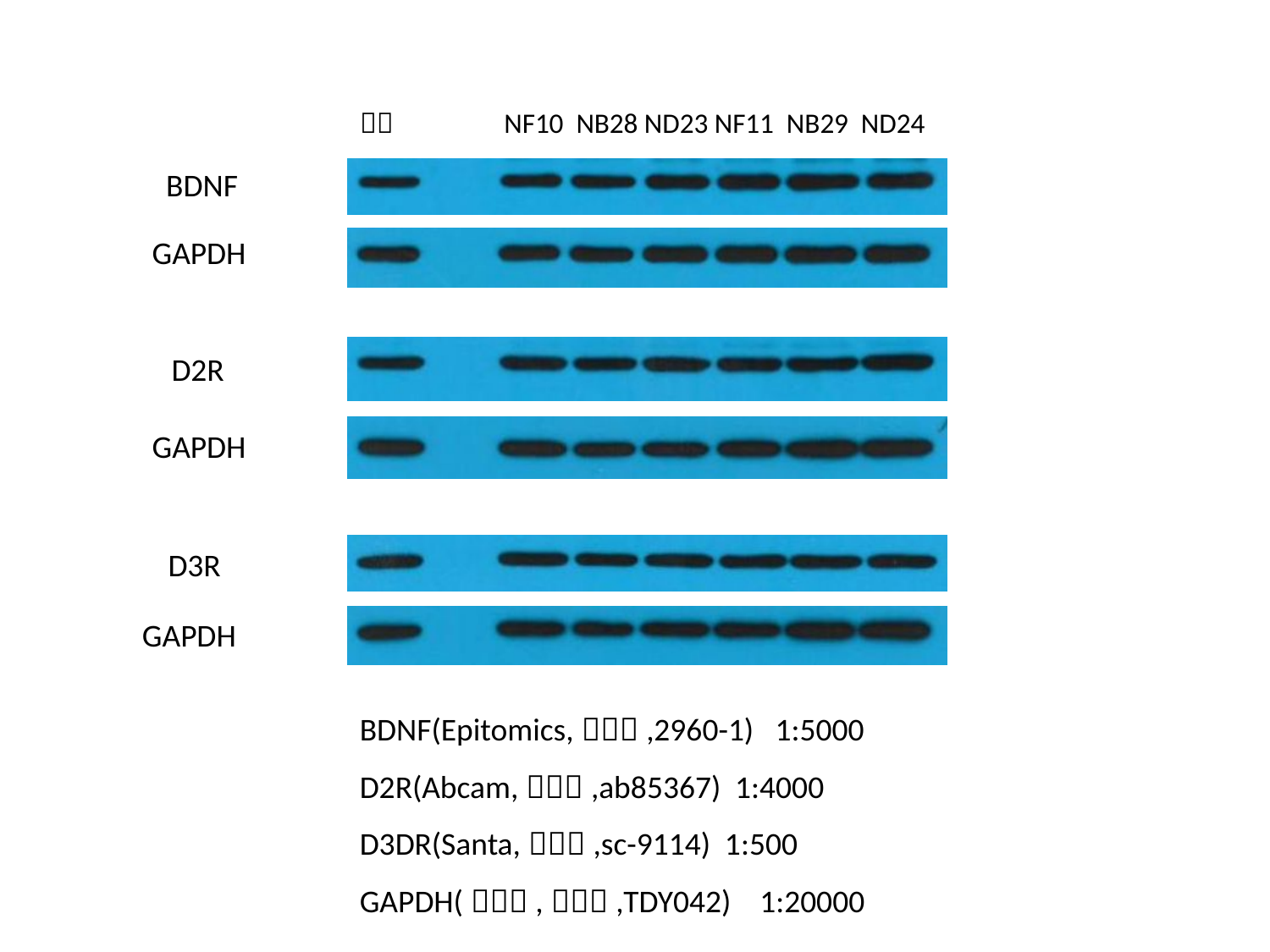

公共 NF10 NB28 ND23 NF11 NB29 ND24
BDNF
GAPDH
D2R
GAPDH
D3R
GAPDH
BDNF(Epitomics,兔单抗,2960-1) 1:5000
D2R(Abcam,兔多抗,ab85367) 1:4000
D3DR(Santa,兔多抗,sc-9114) 1:500
GAPDH(天德悦,鼠单抗,TDY042) 1:20000

## Slide 5
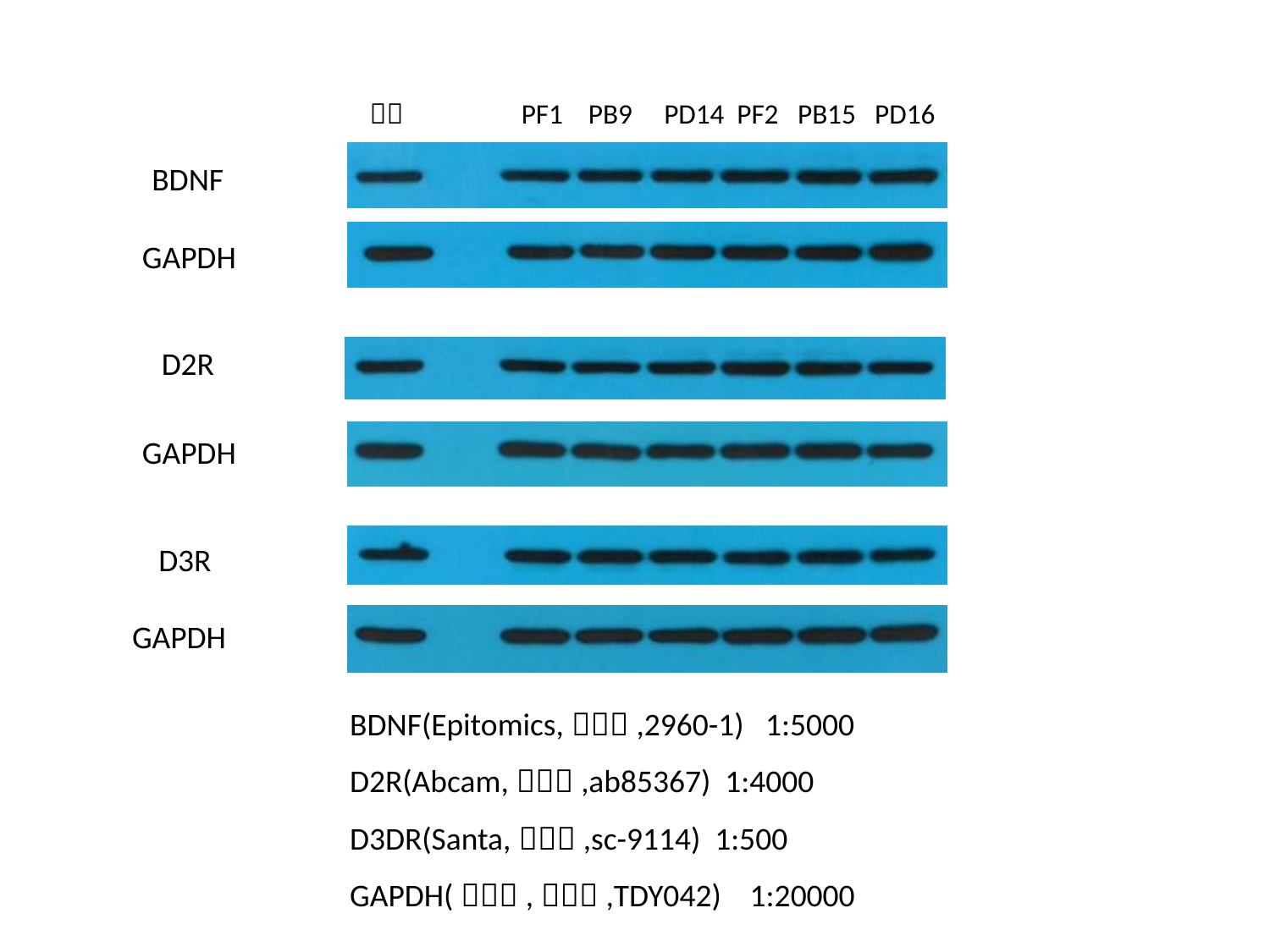

公共 PF1 PB9 PD14 PF2 PB15 PD16
BDNF
GAPDH
D2R
GAPDH
D3R
GAPDH
BDNF(Epitomics,兔单抗,2960-1) 1:5000
D2R(Abcam,兔多抗,ab85367) 1:4000
D3DR(Santa,兔多抗,sc-9114) 1:500
GAPDH(天德悦,鼠单抗,TDY042) 1:20000

## Slide 6
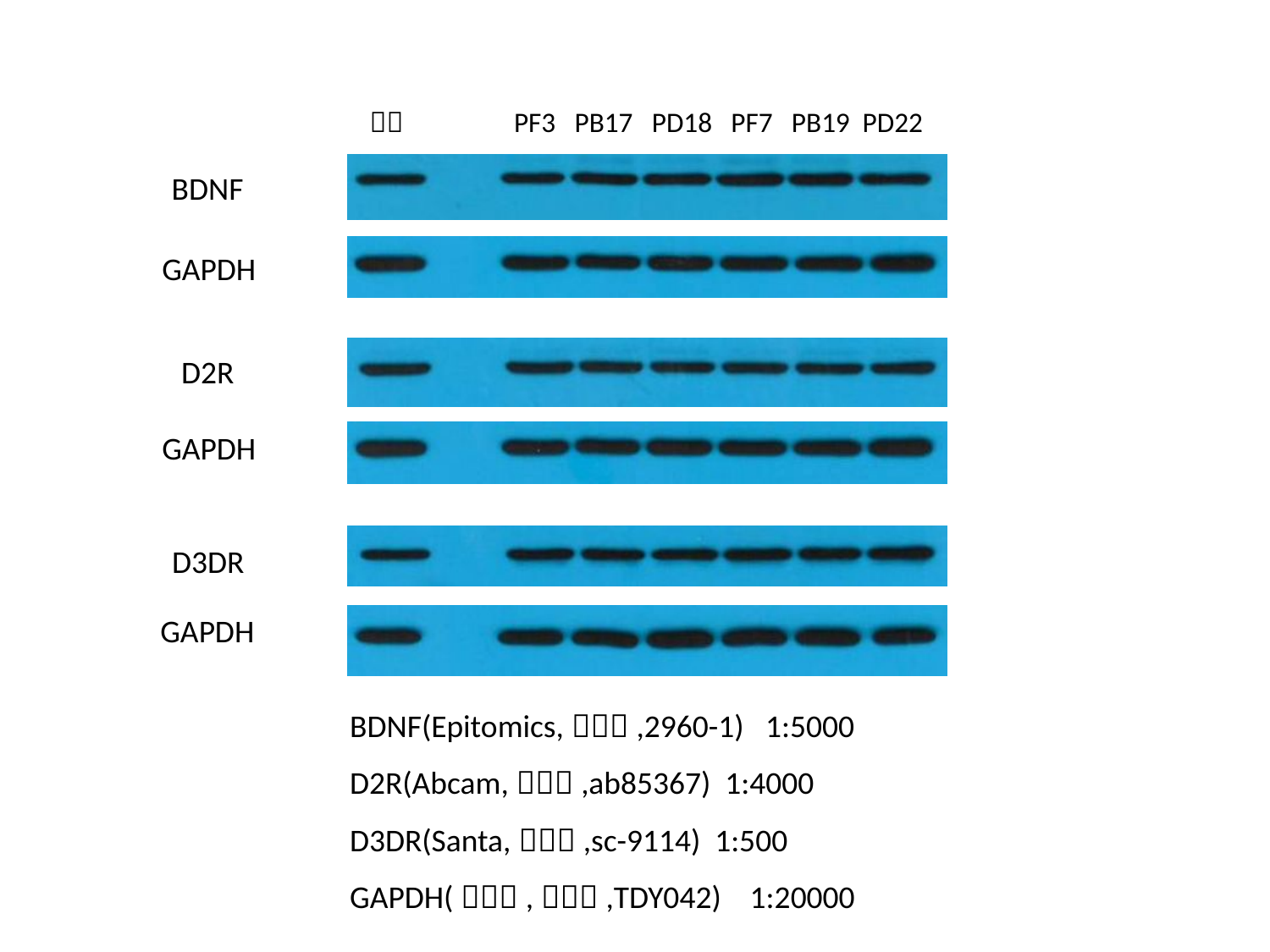

公共 PF3 PB17 PD18 PF7 PB19 PD22
BDNF
GAPDH
D2R
GAPDH
D3DR
GAPDH
BDNF(Epitomics,兔单抗,2960-1) 1:5000
D2R(Abcam,兔多抗,ab85367) 1:4000
D3DR(Santa,兔多抗,sc-9114) 1:500
GAPDH(天德悦,鼠单抗,TDY042) 1:20000

## Slide 7
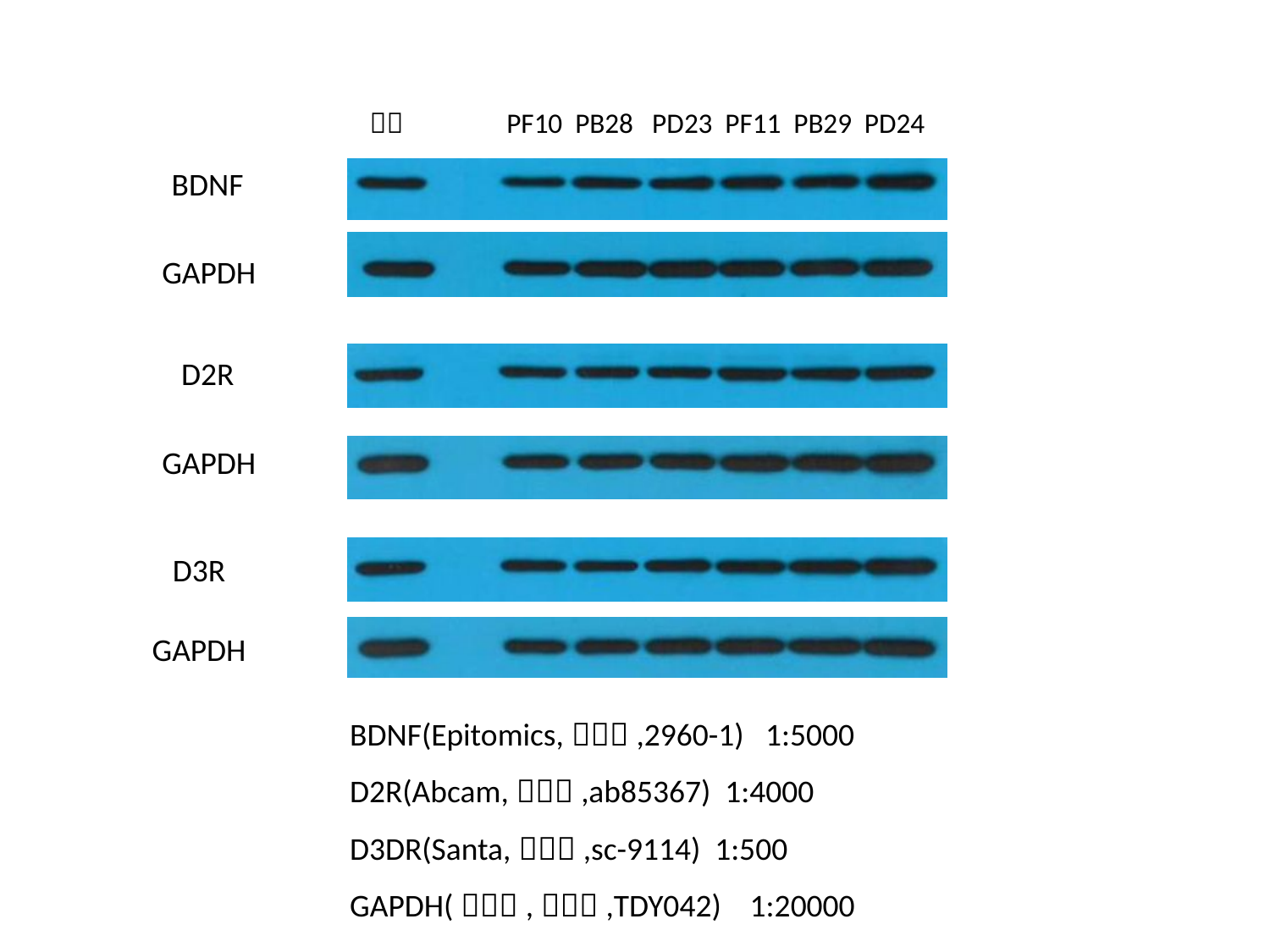

公共 PF10 PB28 PD23 PF11 PB29 PD24
BDNF
GAPDH
D2R
GAPDH
D3R
GAPDH
BDNF(Epitomics,兔单抗,2960-1) 1:5000
D2R(Abcam,兔多抗,ab85367) 1:4000
D3DR(Santa,兔多抗,sc-9114) 1:500
GAPDH(天德悦,鼠单抗,TDY042) 1:20000

## Slide 8
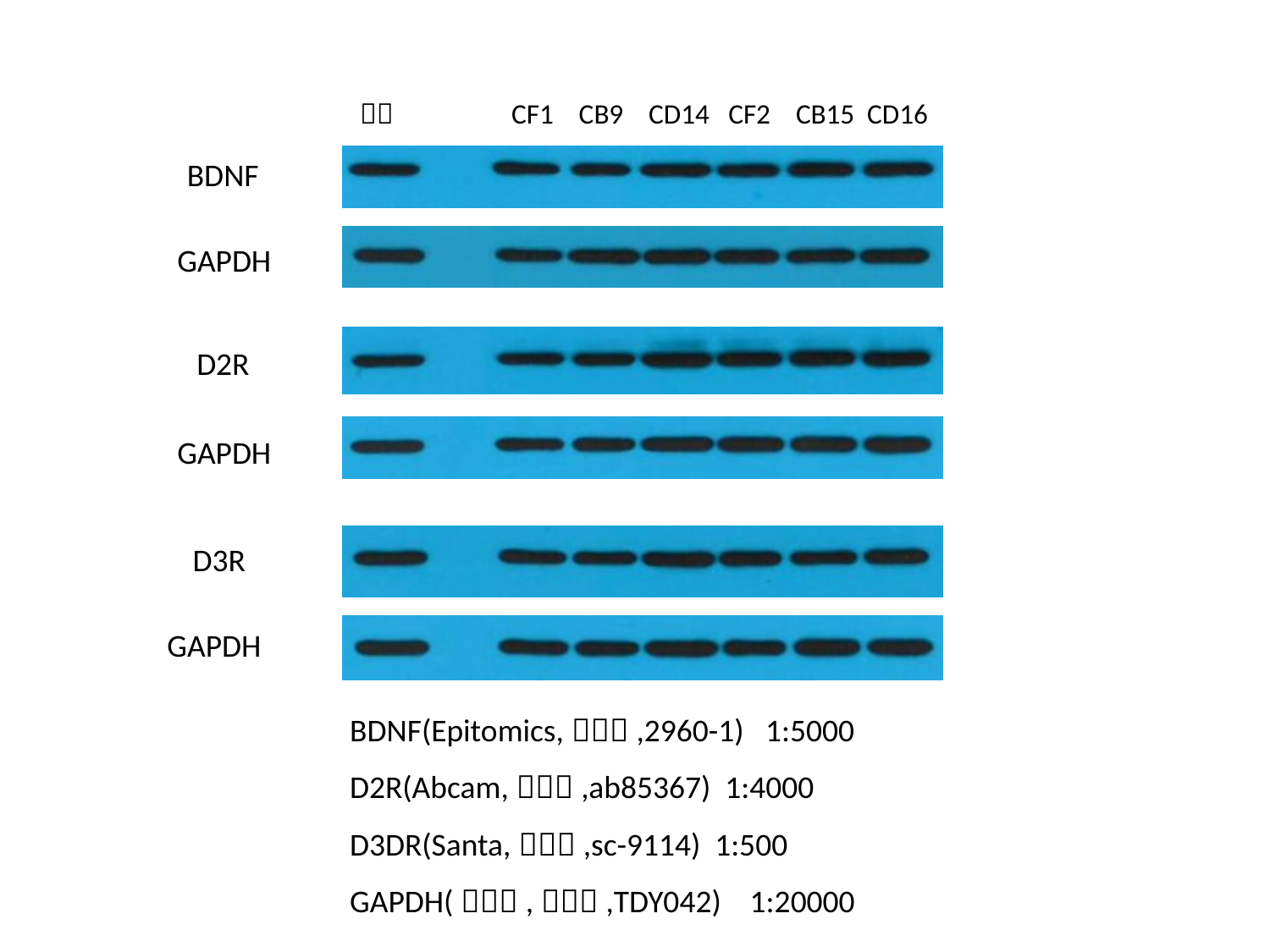

公共 CF1 CB9 CD14 CF2 CB15 CD16
BDNF
GAPDH
D2R
GAPDH
D3R
GAPDH
BDNF(Epitomics,兔单抗,2960-1) 1:5000
D2R(Abcam,兔多抗,ab85367) 1:4000
D3DR(Santa,兔多抗,sc-9114) 1:500
GAPDH(天德悦,鼠单抗,TDY042) 1:20000

## Slide 9
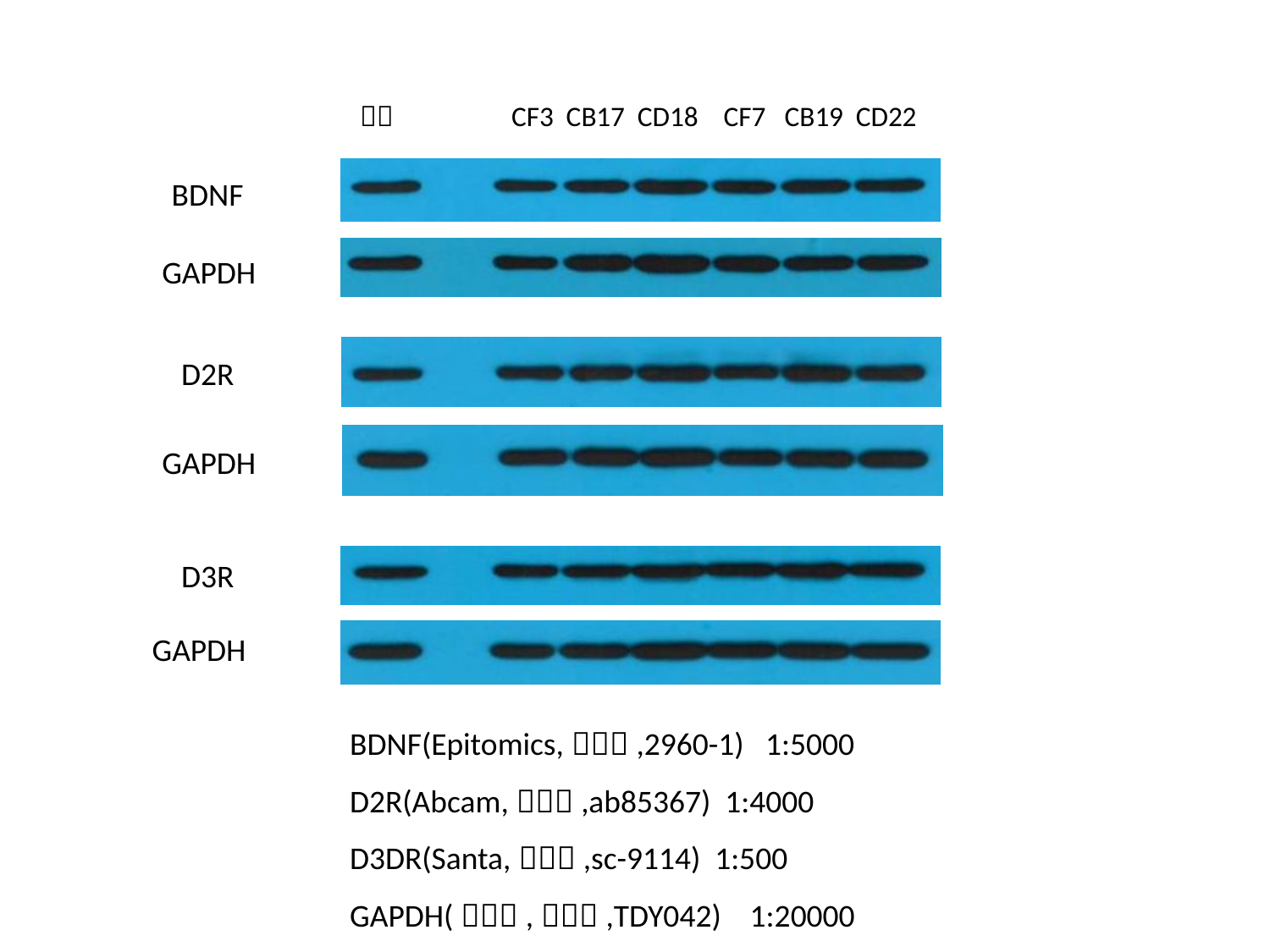

公共 CF3 CB17 CD18 CF7 CB19 CD22
BDNF
GAPDH
D2R
GAPDH
D3R
GAPDH
BDNF(Epitomics,兔单抗,2960-1) 1:5000
D2R(Abcam,兔多抗,ab85367) 1:4000
D3DR(Santa,兔多抗,sc-9114) 1:500
GAPDH(天德悦,鼠单抗,TDY042) 1:20000

## Slide 10
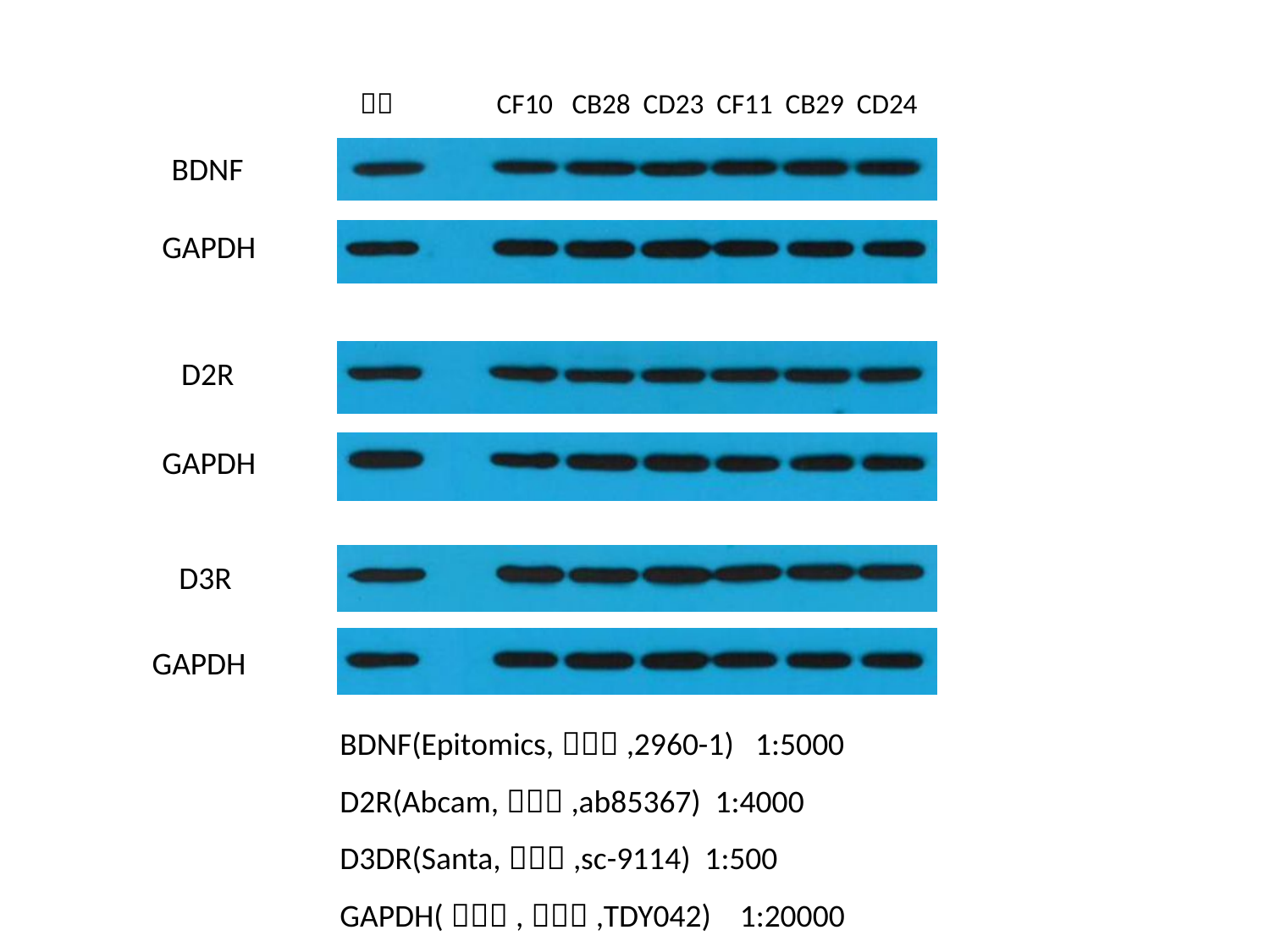

公共 CF10 CB28 CD23 CF11 CB29 CD24
BDNF
GAPDH
D2R
GAPDH
D3R
GAPDH
BDNF(Epitomics,兔单抗,2960-1) 1:5000
D2R(Abcam,兔多抗,ab85367) 1:4000
D3DR(Santa,兔多抗,sc-9114) 1:500
GAPDH(天德悦,鼠单抗,TDY042) 1:20000
