# Supplementary material for: Higher levels of D2R and D3R in the frontal–striatal regions are associated with reduced perseverative reward seeking after opioid abstinence
Source: Front Behav Neurosci. 2025 Jun 2;19:1552055. doi: 10.3389/fnbeh.2025.1552055 (PMC12171296; doi:10.3389/fnbeh.2025.1552055)
Supplement: Supplementary file 2 [file Presentation_2.pptx]

## Slide 1
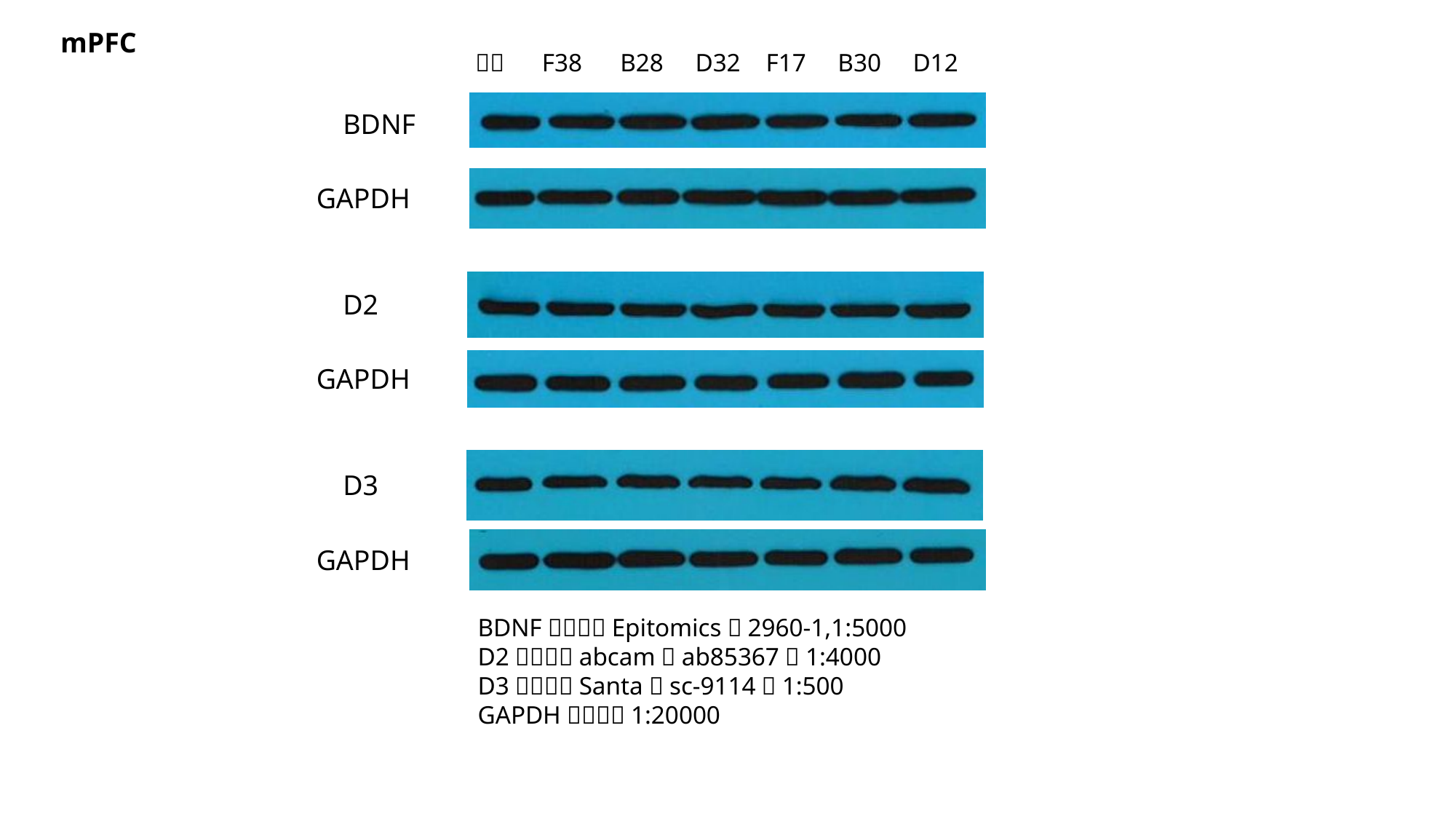

mPFC
公用 F38 B28 D32 F17 B30 D12
BDNF
GAPDH
D2
GAPDH
D3
GAPDH
BDNF兔单抗，Epitomics，2960-1,1:5000
D2兔多抗，abcam，ab85367，1:4000
D3兔多抗，Santa，sc-9114，1:500
GAPDH鼠单抗，1:20000

## Slide 2
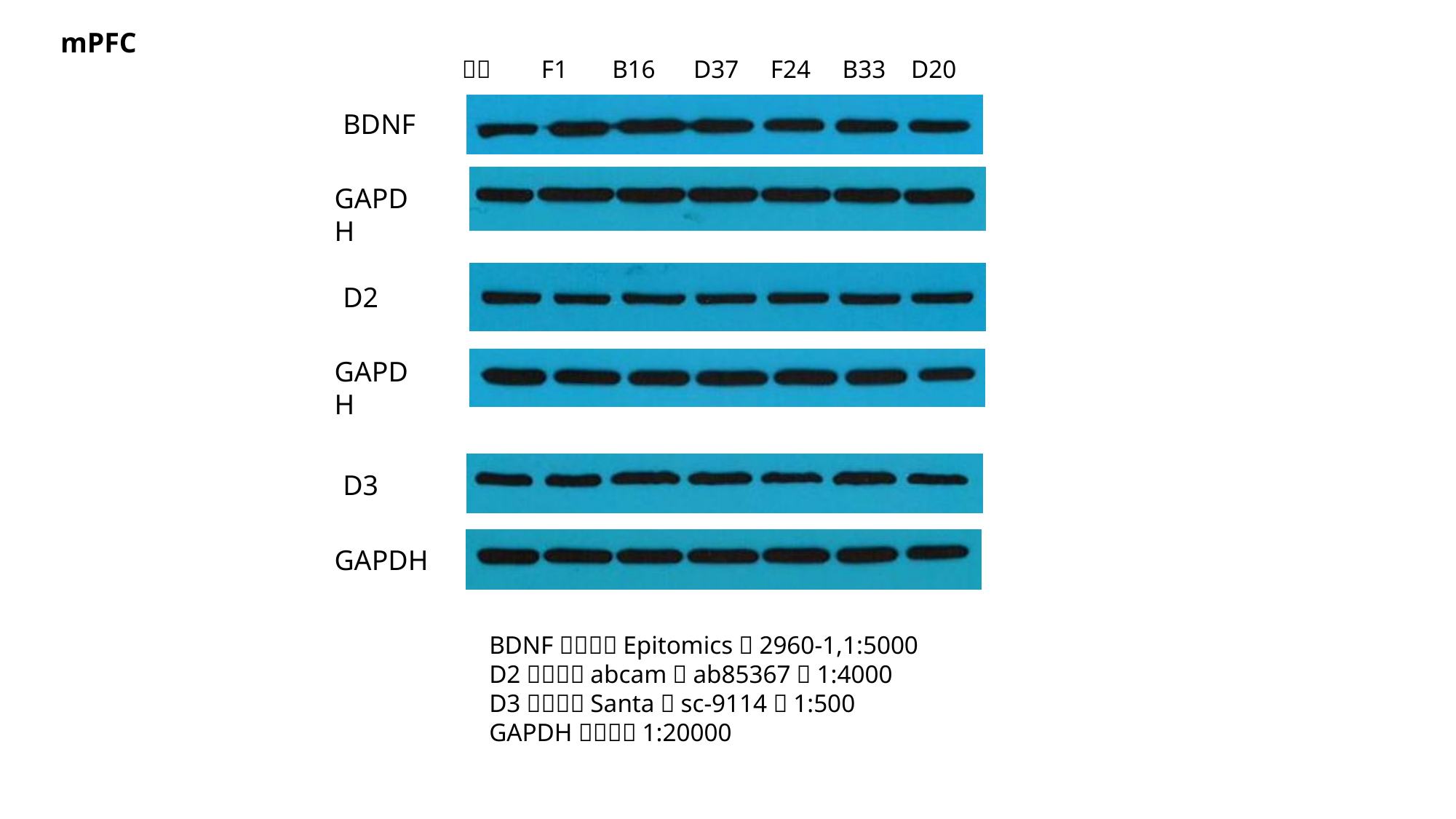

mPFC
公用 F1 B16 D37 F24 B33 D20
BDNF
GAPDH
D2
GAPDH
D3
GAPDH
BDNF兔单抗，Epitomics，2960-1,1:5000
D2兔多抗，abcam，ab85367，1:4000
D3兔多抗，Santa，sc-9114，1:500
GAPDH鼠单抗，1:20000

## Slide 3
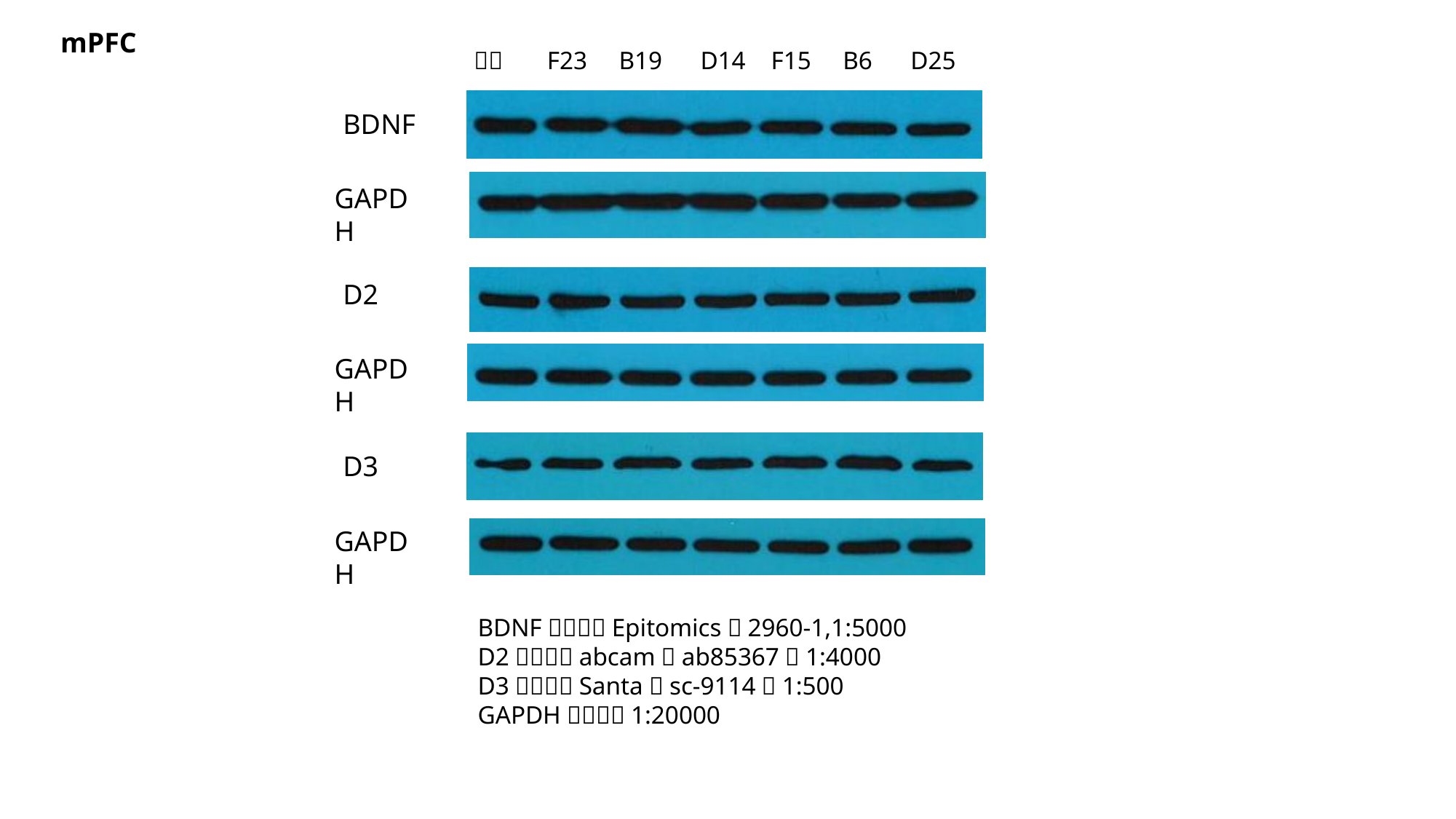

mPFC
公用 F23 B19 D14 F15 B6 D25
BDNF
GAPDH
D2
GAPDH
D3
GAPDH
BDNF兔单抗，Epitomics，2960-1,1:5000
D2兔多抗，abcam，ab85367，1:4000
D3兔多抗，Santa，sc-9114，1:500
GAPDH鼠单抗，1:20000

## Slide 4
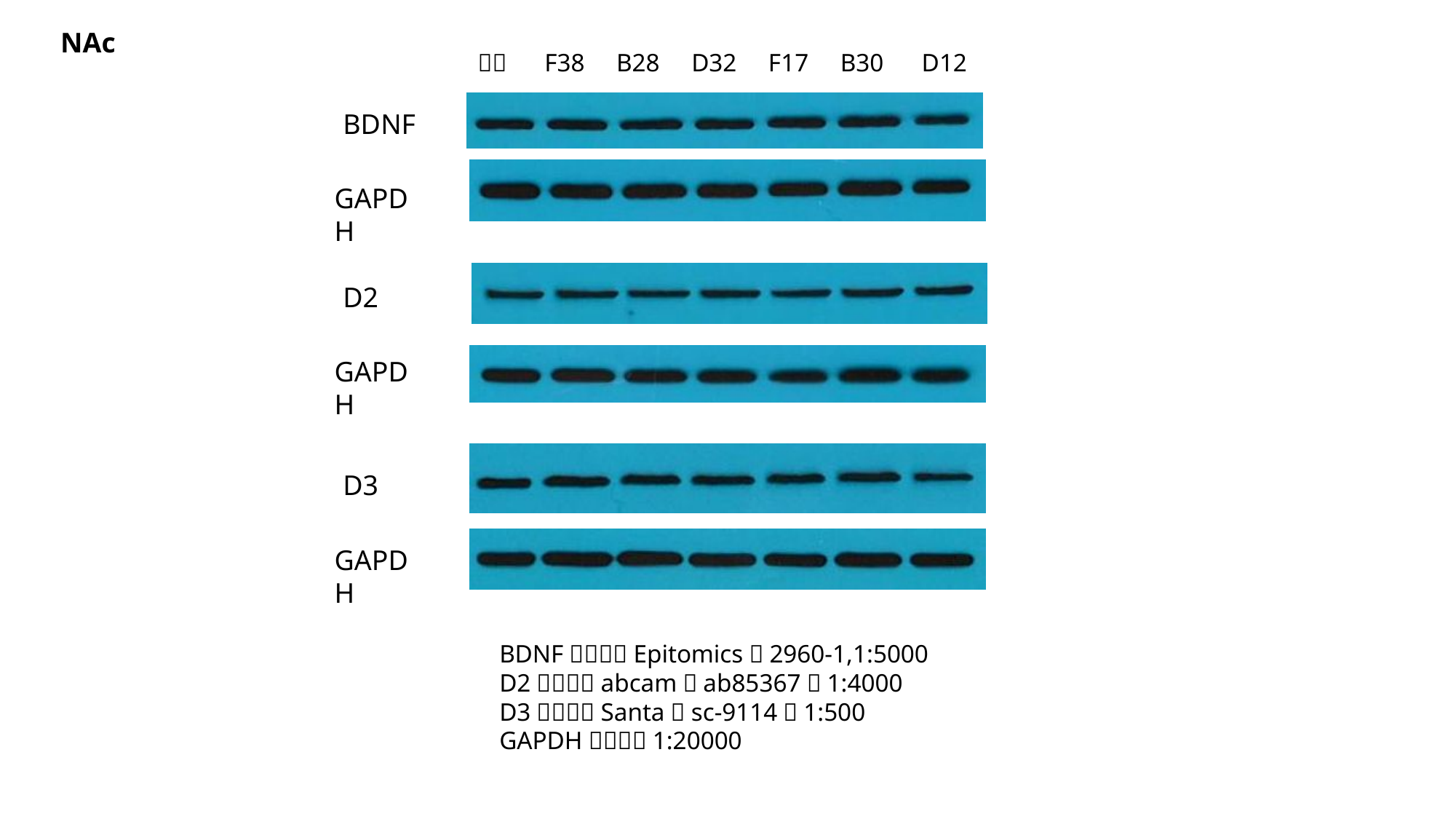

NAc
公用 F38 B28 D32 F17 B30 D12
BDNF
GAPDH
D2
GAPDH
D3
GAPDH
BDNF兔单抗，Epitomics，2960-1,1:5000
D2兔多抗，abcam，ab85367，1:4000
D3兔多抗，Santa，sc-9114，1:500
GAPDH鼠单抗，1:20000

## Slide 5
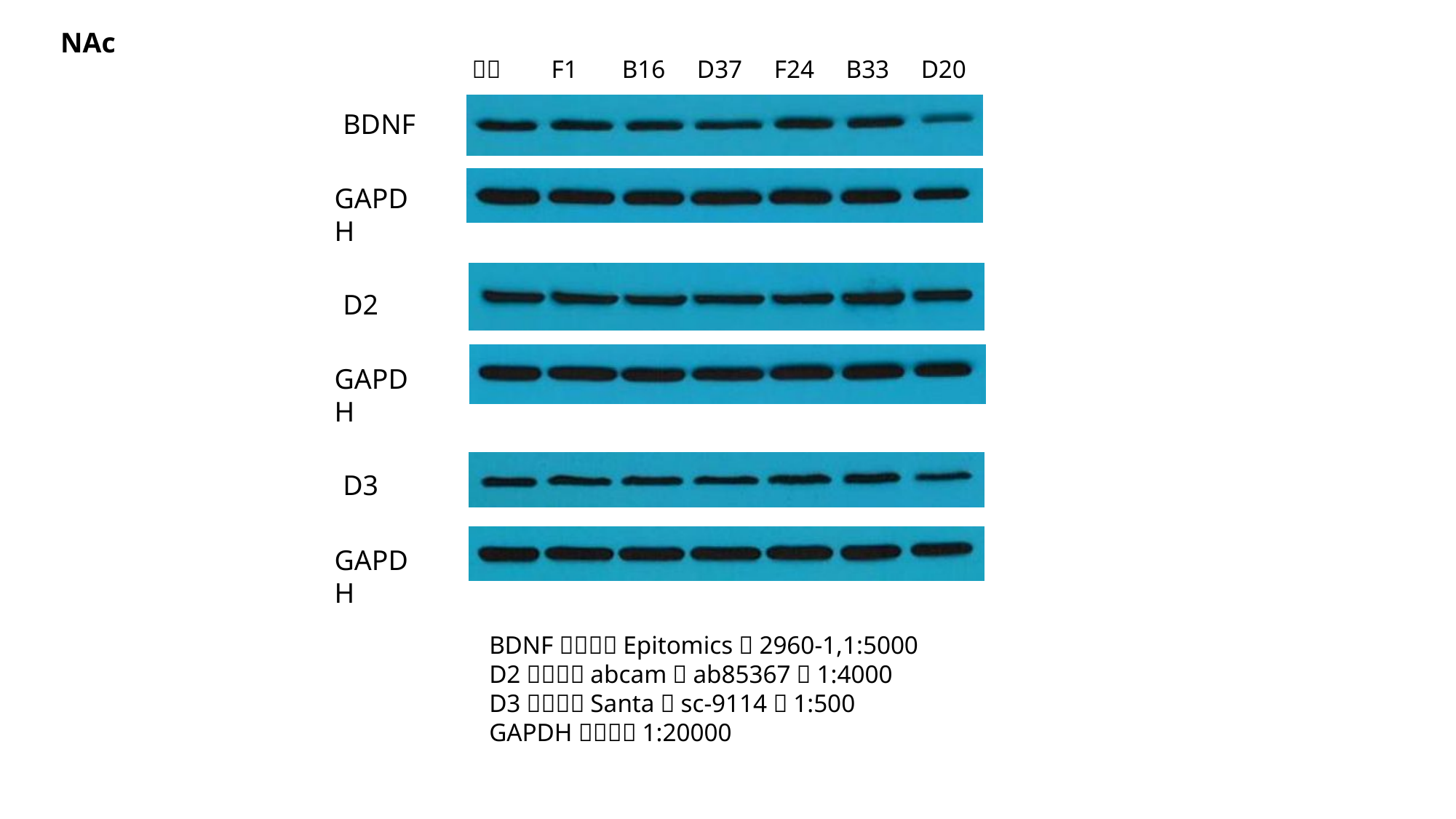

NAc
公用 F1 B16 D37 F24 B33 D20
BDNF
GAPDH
D2
GAPDH
D3
GAPDH
BDNF兔单抗，Epitomics，2960-1,1:5000
D2兔多抗，abcam，ab85367，1:4000
D3兔多抗，Santa，sc-9114，1:500
GAPDH鼠单抗，1:20000

## Slide 6
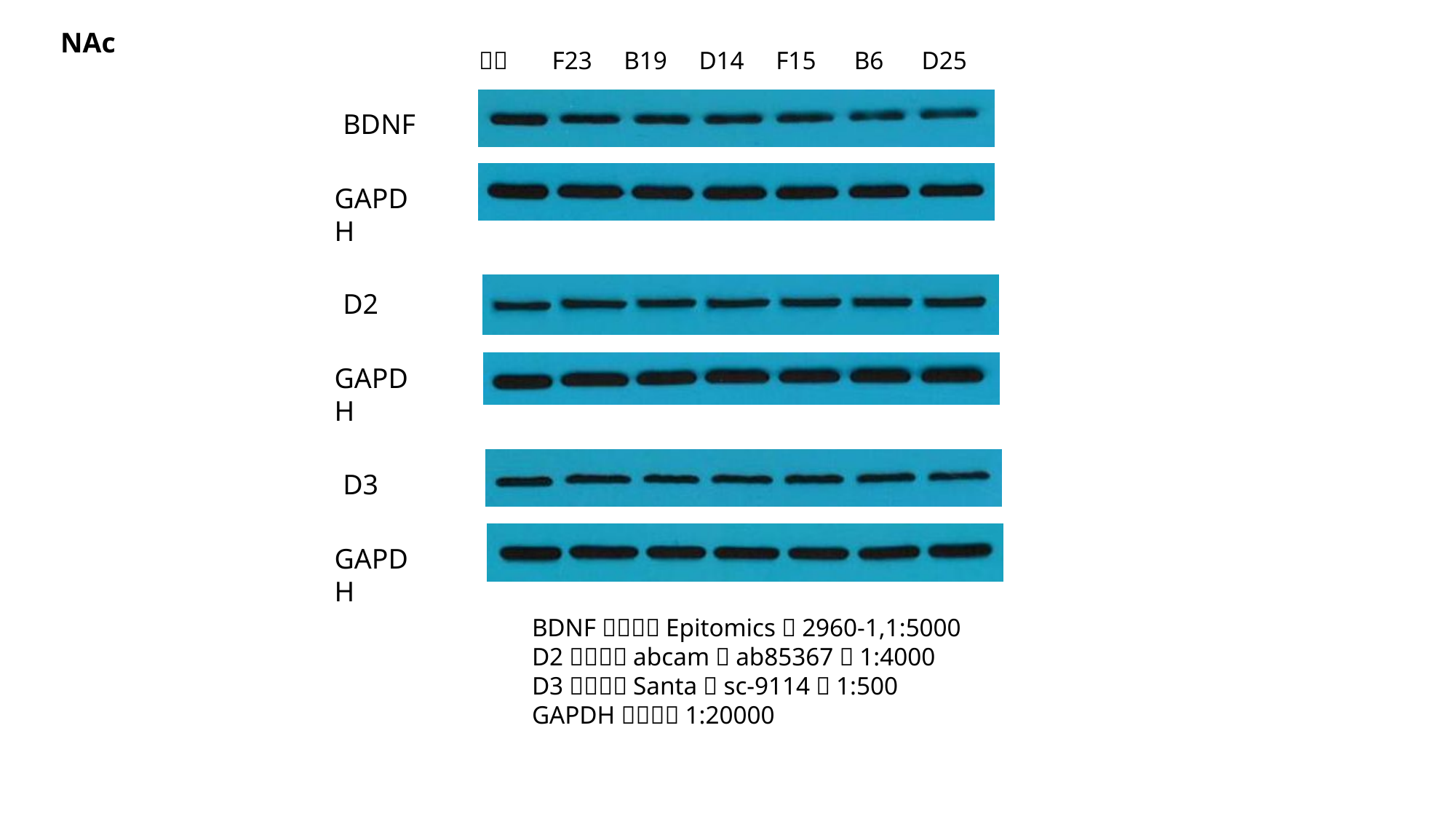

NAc
公用 F23 B19 D14 F15 B6 D25
BDNF
GAPDH
D2
GAPDH
D3
GAPDH
BDNF兔单抗，Epitomics，2960-1,1:5000
D2兔多抗，abcam，ab85367，1:4000
D3兔多抗，Santa，sc-9114，1:500
GAPDH鼠单抗，1:20000

## Slide 7
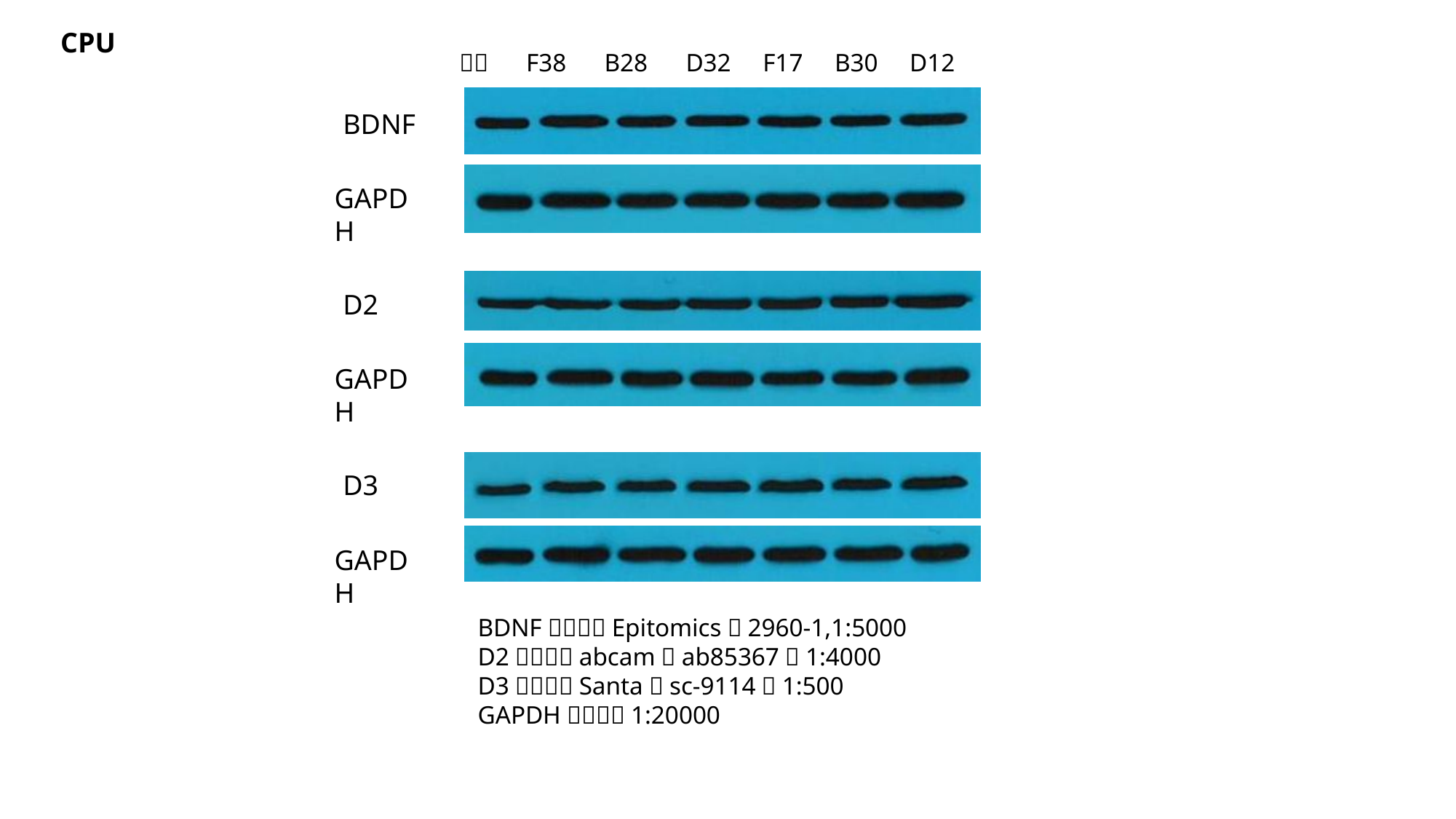

CPU
公用 F38 B28 D32 F17 B30 D12
BDNF
GAPDH
D2
GAPDH
D3
GAPDH
BDNF兔单抗，Epitomics，2960-1,1:5000
D2兔多抗，abcam，ab85367，1:4000
D3兔多抗，Santa，sc-9114，1:500
GAPDH鼠单抗，1:20000

## Slide 8
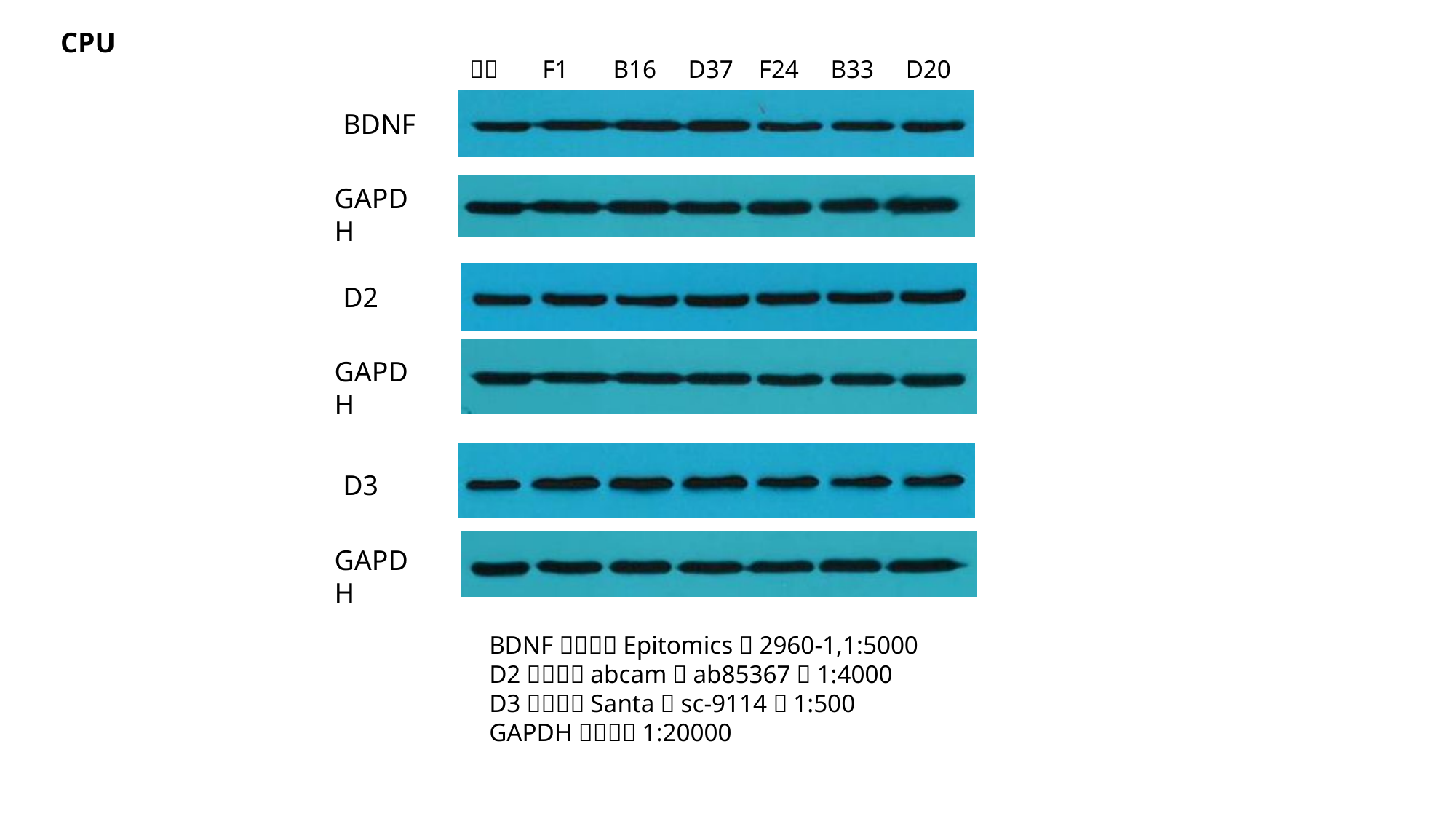

CPU
公用 F1 B16 D37 F24 B33 D20
BDNF
GAPDH
D2
GAPDH
D3
GAPDH
BDNF兔单抗，Epitomics，2960-1,1:5000
D2兔多抗，abcam，ab85367，1:4000
D3兔多抗，Santa，sc-9114，1:500
GAPDH鼠单抗，1:20000

## Slide 9
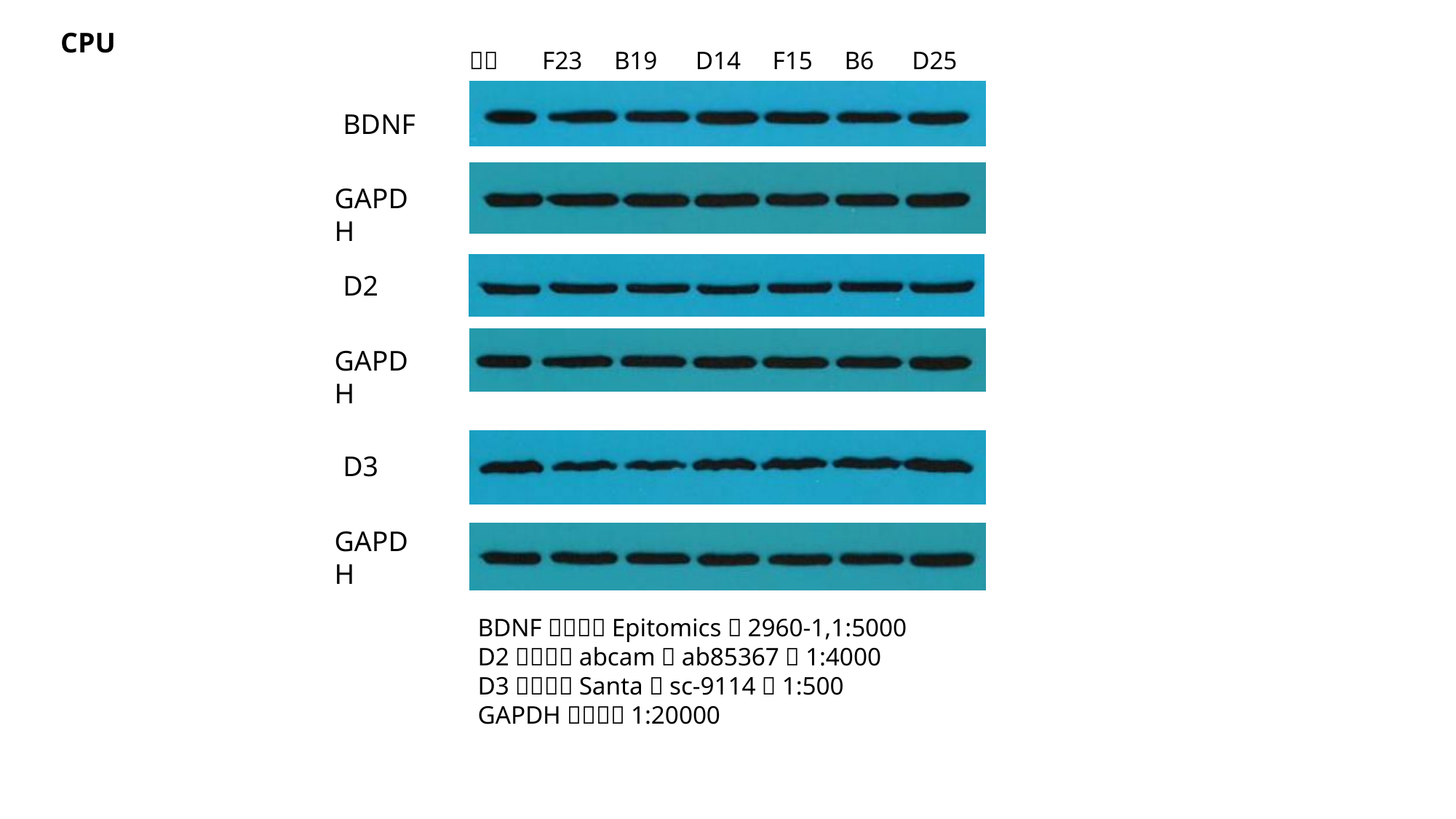

CPU
公用 F23 B19 D14 F15 B6 D25
BDNF
GAPDH
D2
GAPDH
D3
GAPDH
BDNF兔单抗，Epitomics，2960-1,1:5000
D2兔多抗，abcam，ab85367，1:4000
D3兔多抗，Santa，sc-9114，1:500
GAPDH鼠单抗，1:20000
