# Supplementary figures and images for: Higher levels of D2R and D3R in the frontal–striatal regions are associated with reduced perseverative reward seeking after opioid abstinence
Source: Front Behav Neurosci. 2025 Jun 2;19:1552055. doi: 10.3389/fnbeh.2025.1552055 (PMC12171296; doi:10.3389/fnbeh.2025.1552055)

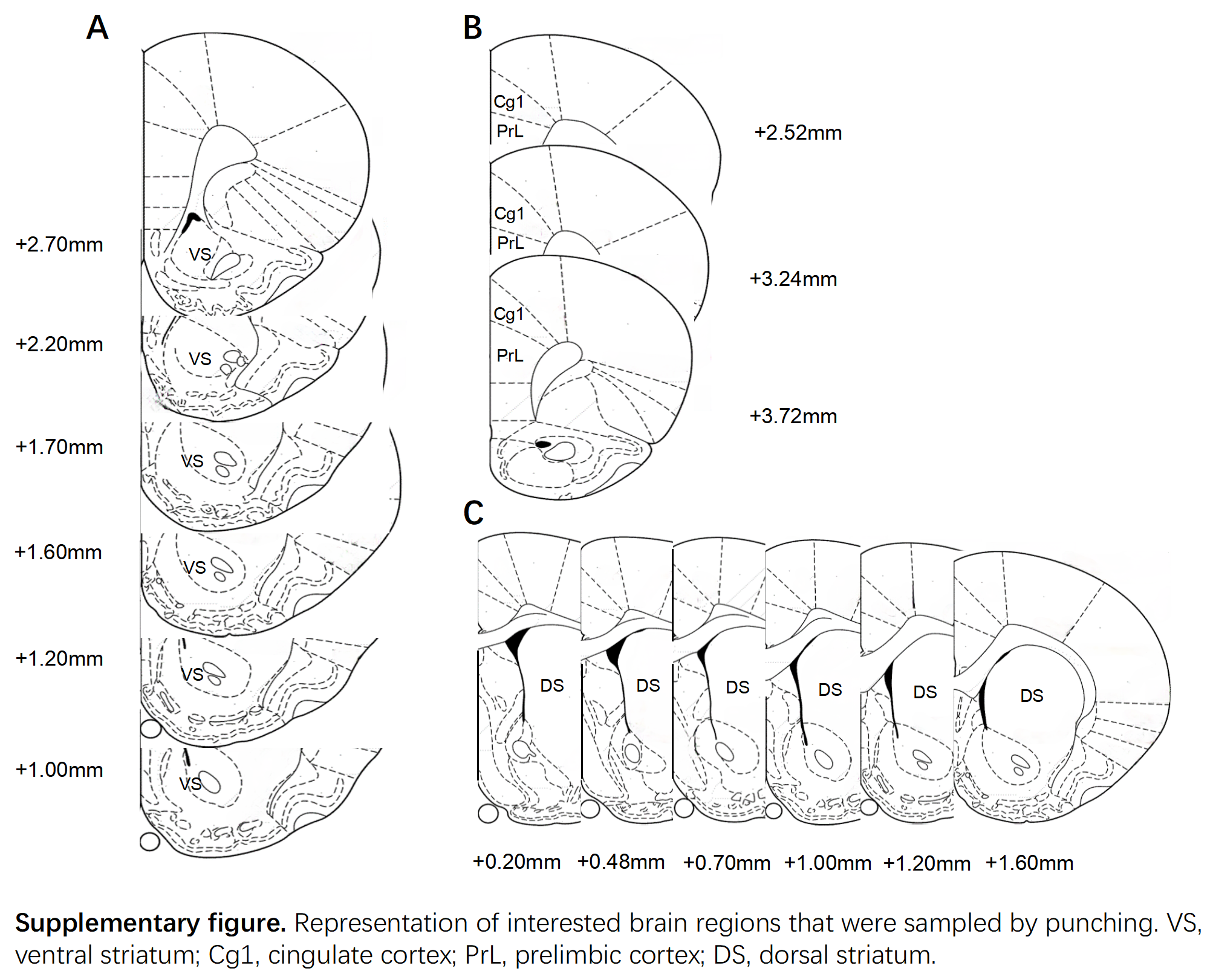

Supplement: Supplementary file 4 [file Image_1.tif]
